# FUT8 upregulates CD36 and its core fucosylation to accelerate pericyte-myofibroblast transition through the mitochondrial-dependent apoptosis pathway during AKI-CKD

**DOI:** 10.1186/s10020-024-00994-6

**Published:** 2024-11-20

**Authors:** Yaxi Shang, Ziran Wang, Fan Yang, Weidong Wang, Qingzhu Tang, Xianan Guo, Xiangning Du, Xu Zhang, Jiaojiao Hao, Hongli Lin

**Affiliations:** Department of Nephrology, The First Affiliated Hospital of Dalian Medical University, Dalian, Liaoning Province 116011 PR China

**Keywords:** AKI-CKD, Core fucosylation, CD36, FUT8, Pericyte

## Abstract

**Background:**

Activation of pericytes leads to renal interstitial fibrosis, but the regulatory mechanism of pericytes in the progression from AKI to CKD remains poorly understood. CD36 activation plays a role in the progression of CKD. However, the significance of CD36 during AKI-CKD, especially in pericyte, remains to be fully defined.

**Methods:**

GEO and DISCO database were used to analyze the expression of CD36 in pericyte during AKI-CKD; IRI to conduct AKI-CKD mouse model; Hypoxia/Reoxygenation (H/R) to induce the cell model; RT-qPCR and Western blotting to detect gene expression; IP and confocal-IF to determine the core fucosylation (CF) level of CD36. Flow cytometry (AV/PI staining) to detect the cell apoptosis and JC-1 staining to react to the change of mitochondrial membrane potential.

**Results:**

During AKI to CKD progression, CD36 expression in pericytes is higher and may be influenced by CF. Moreover, we confirmed the positive association of CD36 expression with pericyte-myofibroblast transition and the progression of AKI-CKD in an IRI mouse model and hypoxia/reoxygenation (H/R) pericytes. Notably, we discovered that FUT8 upregulates both CD36 expression and its CF level, contributing to the activation of the mitochondrial-dependent apoptosis signaling pathway in pericytes, ultimately leading to the progression of AKI-CKD.

**Conclusion:**

These results further identify FUT8 and CD36 as potential targets for the treatment in the progression of AKI-CKD.

**Supplementary Information:**

The online version contains supplementary material available at 10.1186/s10020-024-00994-6.

## Introduction

Acute kidney injury (AKI) is a pathological syndrome described by a sudden decline in kidney function, which results in approximately 13 million individuals being affected globally and causes nearly 2 million deaths annually (Singbartl and Kellum 2012). Survivors of AKI face a high risk of chronic kidney disease (CKD) and even end-stage renal disease (ESRD), resulting in a marked increase in morbidity and mortality, thus representing a global health concern (Hull et al. 2017; Kellum et al. 2021). Although the disrupted cellular processes and their variable evolutions during AKI-CKD have been exhaustively investigated, there is still an urgent need to better understand the endogenous factors involved in AKI-CKD.

Renal pericytes, a diverse population derived from the mesenchyme, attach to the endothelial cells of blood vessels, providing vascular stability (Kim et al. 2024). Activation of pericytes leads to destabilization of the vessel walls, causing peritubular capillary loss, tissue ischemia, and hypoxia, thereby exacerbating renal fibrosis (Tanaka et al. 2023). Research has shown that myofibroblasts are mainly derived from pericytes and fibroblasts (Kuppe et al. 2021). Despite being the main “culprit” cells in the development of renal interstitial fibrosis, the role of pericyte injury in the progression from AKI to CKD remains poorly understood.

Glycosylation, as a post-translational modification, plays a vital role in the progress of kidney diseases (Dotz et al. 2021; Suzuki and Novak 2021; Chen and Gan 2023). Core fucosylation (CF) is considered an essential method of post-translational processing and the functional regulation of glycoproteins (Wang et al. 2024). α1,6 Fucosyltransferase (FUT8) is a unique enzyme that catalyzes the CF modification of N glycans and has been certified to play significant roles in signal transduction and immune regulation during kidney injury (Zhang et al. 2023; Shen et al. 2013; Venkatachalam and Weinberg 2013; Liu et al. 2022). Research has shown that increased levels of FUT8-mediated CF are associated with renal interstitial fibrosis (RIF), inhibiting FUT8-mediated CF can mitigate RIF, indicating its potential as a therapeutic target (Shen et al. 2013; Li et al. 2018). However, the specific molecular mechanisms by which FUT8 regulates the progression from AKI to CKD remain to be fully elucidated. Our previous study found that knockout of FUT8 in renal tubular epithelial cells not only reduced their own damage, but also possibly downregulated CF levels in surrounding endothelial cells and pericytes through paracrine signaling, thereby reducing damage to these cells (Li et al. 2023). Additionally, as a key type of cell death, apoptosis induced by mitochondrial dysfunction plays an exceptional role in the progression from AKI to CKD (Aranda-Rivera et al. 2021), including tubular cell regeneration and interstitial fibrosis (Zhang et al. 2021); however, the mechanism by which FUT8 regulates this pathway is still unknown. Collectively, the role of FUT8 in pericytes requires further exploration. Identifying these mechanisms could develop novel therapeutic strategies that target FUT8 to prevent or reduce kidney damage in the progression from AKI to CKD.

Platelet glycoprotein 4 (CD36), a scavenger receptor, is crucial to mediate lipid uptake and contributes significantly to mitochondrial damage in the context of kidney diseases (Ma et al. 2024). Activation of CD36 in renal cells has been demonstrated to initiate inflammatory pathways, resulting in the generation of pro-inflammatory cytokines and reactive oxygen species (ROS) and ultimately leading to cellular damage and apoptosis (Yang et al. 2017). These responses exacerbate kidney damage and fibrosis, having a role in the progression of CKD (Okamura et al. 2009). Targeting CD36 to reduce lipid uptake and mitigate its downstream effects on mitochondrial function represents a potential therapeutic strategy. It was also reported that glycosylation of CD36 is crucial for its function in lipid metabolism and inflammatory signaling (Okamura et al. 2009), significantly impacting the pathogenesis of kidney diseases (Demers et al. 2004; Zou et al. 2023). However, the functional significance of CD36 during AKI-CKD remains to be fully defined.

Herein, we used bioinformatic analysis of transcriptome data to show that CD36 expression is elevated during AKI to CKD progression and might be modified by CF. Single-cell sequencing analysis also revealed that CD36 is highly expressed in pericytes. In addition, we identified that pericyte CD36 could potentially facilitate pericyte transition and contribute to renal fibrosis during the progression of AKI-CKD. Notably, we discovered that FUT8 not only regulates the expression of CD36 in pericytes, but also influences its core fucosylation levels, and this regulation leads to mitochondrial damage-induced apoptosis, thereby promoting the transition from AKI to CKD. These results further identify FUT8 and CD36 as potential targets for the treatment in the progression of AKI-CKD. The precision medicine therapy focusing on FUT8/CD36 holds significant promise for patients suffering from AKI on a global scale.

## Materials and methods

### Ethics statement

To ensure that animal experimental protocols adhered to scientific principles and humanity, all experiments using mice in vivo were performed with the supervision and approval of the Animal Ethics Committee of Dalian Medical University. (Doc. No. AEE24009)

### Collection and analysis of data

The GSE140988 dataset, a gene expression data focusing on pericytes from mice kidneys before or on day 2,7 and 28 after acute kidney injury, was sourced from the gene expression omnibus (GEO) database (https://www.ncbi.nlm.nih.gov/geo/). Fold changes of gene expression after normalization were calculated through the “limma” R package (R Core Team. 2020). Single-cell RNA sequencing data of kidney tissues was acquired and analyzed via the deeply integrated human single-cell omics database (DISCO) database (https://www.immunesinglecell.org).

### Animals and renal ischemia-reperfusion injury model

Wild-type male C57BL/6 mice aged 8–12 weeks and weighing 20–30 g were purchased from Liaoning Changsheng Biotechnology Co., Ltd. (Shenyang, China), they were all placed in a mouse-friendly environment with standard rodent chow and water provided by the Renal Pathology Center of the First Affiliated Hospital of Dalian Medical University. Animal surgeries were performed by the same surgeon under sterile conditions with most efforts made to reduce the suffering of animals. On a constant temperature blanket, the mice were induced by clamping bilateral renal pedicles for 55 min via a dorsal incision under sodium pentobarbital anesthesia to establish the acute kidney injury model. Mice were divided into Sham, ischemia-reperfusion injury (IRI) 2D, IRI 7D, and IRI 30D groups. The mice in the sham group only underwent skin and muscle incisions without renal pedicle clamping. Renal tissues and sera were collected subsequently.

### Histological analysis

The harvested mouse kidneys were put in 4% paraformaldehyde, dehydrated, and packed in paraffin. Paraffin-embedded tissue was cut into slices using a paraffin slicer and attached to glass slides. Each section was first dewaxed and dehydrated with xylene and ethanol before staining with Masson’s Trichrome and Periodic Acid Schiff (PAS). Image acquisition was scanned on a 3DHISTECH Pannoramic Scanner (3DHISTECH, Budapest, Hungary).

### Immunohistochemical analysis

Sections first underwent dewaxing and hydration, followed by antigen retrieval and quenching of endogenous peroxidase activity. After clearing in 0.01 M phosphate-buffered saline (PBS) and incubating in blocking buffer for 20 min, the primary antibodies were trickled to the sections and placed for one night at 4 ℃. Appropriate secondary antibodies were then applied. The color was developed using the substrate diaminobenzidine. Finally, dehydration and purification of slices were performed in 95% alcohol and xylene. Prepared sections were sealed in neutral gum for image acquisition.

### Immunofluorescence (IF) staining

Paraffin sections were dewaxed and subjected to antigen retrieval in a microwave with citrate buffer (0.01 M citrate buffer, pH 6.0). At the same time, cells in twelve-well plates were incubated for 5 minutes in 0.5% Triton X 100 solution (Sigma, St. Louis, MO, USA) after being fixed in 4% paraformaldehyde for 30 min at 25℃. All samples were soaked with 10% blocking goat serum in PBS for 30 min at 25℃ before primary antibodies incubation at 4℃. The sections were returned to room temperature and added some fluorescently-labeled secondary antibodies next day. 4’,6-diamidino-2-phenylindole (DAPI) was used to label cell nuclei at the end. Photographs were captured using a confocal laser scanning microscope (SP8, Leica, Wetzlar, Germany).

### Pericyte extraction from kidney tissue

Pericytes were extracted from C57/BL6 wild-type male mouse kidneys through dicing and digestion with liberase (0.5 mg/mL, Roche, Mannheim, Germany) and DNase (100U/mL, Roche) in Hank’s buffered salt solution at 37 °C for 45 min. Cells which were centrifuged and resuspended with Hank’s buffered salt solution were filtered (40 μm) and purified by gradient using a 42% Percoll solution. Then platelet derived growth factor receptor beta + (PDGFRβ+) cells were picked with the help of fluorescence-activated cell sorting (FACS) (FACSAria cell sorting, BD Biosciences, San Jose, CA, USA). Finally, pericytes were cultured in Dulbecco’s modified Eagle’s medium (DMEM)-F12 (Gibco Life Technologies, Grand Island, NY, USA) supplemented with 10% fetal bovine serum (FBS) and 1% penicillin/streptomycin (Gibco Life Technologies) in a normal incubator (37 °C, 5% CO2 atmosphere, 90% humidity). Pericytes in the first passage were used for the experiments. Pericytes cultured without FBS were subjected to simulated ischemia-reperfusion injury in vivo by exposure to hypoxic conditions in a hypoxic incubator for 24 h (94% N2, 5% CO2, and 1% O2) and reoxygenation for an additional 6–72 h.

### SiRNA and overexpression (OE) plasmid transfection

Pericytes were transfected with designed CD36 and FUT8 siRNA or negative controls (NC) (Unibio, Chongqing, China) and overexpression or GFP (Green fluorescent protein) plasmid using lipofectamine 3000 reagents (L3000015, Invitrogen, Waltham, MA, USA). Briefly, cells were cultured in a complete DMEM/F12 medium without penicillin/streptomycin. Upon reaching 50% confluence, the Opti-MEM medium (31985070, Gibco) with the siRNA or the overexpression plasmid and 5µL of lipofectamine 3000 reagents was mixed into the cells. 6 h later, pericytes were switched back to complete DMEM/F12 medium and grown to 80% confluence.

### Transmission electron microscopy (TEM)

TEM was employed to further detect mitochondrial morphology. Pericytes harvested by 0.25% trypsinization were soaked in 2.5% glutaraldehyde at 4 °C for at least one night and then incubated with 1% osmic acid for 2 h at 4 °C. Subsequently the dehydrated pericytes were embedded in EPON 812 (14130, Electron Microscopy Sciences, Hatfield, PA, USA) and sectioned into ultrathin slices which were stained with uranyl acetate dihydrate and lead citrate. Images were performed on a TEM (1400plus, Hitachi, Tokyo Japan).

### Flow cytometry analysis

An annexin V alexa fluor647(AV)/propidium iodide (PI)/Apoptosis detection kit (KGA1101-100, KeyGENBioTECH, Nanjing, China) for flow cytometry was used to detect apoptotic pericytes. In short, cells were prepared by washing, digesting, and resuspension in binding buffer, followed by replenishment with 5 µL of Annexin V and 5 µL of PI for 15 min on ice in the dark room. At last, a 200ul binding buffer was added. The final inspection is carried out on a Navios Flow cytometer (Beckman Coulter, Indianapolis, IN, USA). Data were analyzed with FlowJo software (TreeStar, Ashland, OR, USA).

### Deglycosylation for N-glycan analysis

According to the instructions of the N-glycosidase F kit (GE10001, Glpbio, Montclair, CA, USA), 10% NP-40 and N-glycosidase F (PNGase F) were added to pericyte lysates at 2 µL per 50 µg of cell lysates and incubated for 3 h at 37 °C. Then, the deglycosylated lysates were used for western blotting (WB) using corresponding antibodies to assess the effects of deglycosylation.

### Real-time fluorescence quantitative reverse transcription PCR (RT-qPCR)

Total RNA was extracted from pericytes according to the TRIzol reagent’s (Invitrogen) protocol. Reverse transcription was performed by the FastKing cDNA First Strand Synthesis Kit (Genome-free) (TIANGEN, Beijing, China). The real-time quantitative polymerase chain reaction was carried out with the help of a Talent Fluorescent Quantitative PCR Kit (SYBR Green) (TIANGEN) on the 7500 FAST Real-Time PCR system (Thermo Fisher Scientific, Waltham, MA, USA). Relative CD36 expression was normalized to glyceraldehyde-3-phosphate dehydrogenase (GAPDH) expression using the ΔΔCt method (Livak and Schmittgen 2001). The several primer sequences used were: CD36, F5′-ACAGTCTCTTTCCTGCAGCC-3′; R5′-CTGCCACAGCCAGATTGAGA-3′; GAPDH, F5′-AGGTCGGTGTGAACGGATTTG-3′; R5’-TGTAGACCATGTAGTTGAGGTCA-3′.

### Western blotting

Protein expression in kidneys and pericytes was detected by western blotting. Cell or tissue lysates were denatured by heating at 99 °C for 5–10 min with sodium dodecyl sulfate-polyacrylamide gel electrophoresis (SDS-PAGE) loading buffer. The separated protein on 10% SDS-PAGE gels was transferred onto polyvinylidene fluoride membranes. After blocking with gelatin blocking solution in PBST (PBS with 0.1% Tween20) buffer for 2 h at 25 °C. Every membrane was probed with appropriate primary antibodies at 4 °C for 12 h. Afterwards, excess solution on the membranes was washed three times for 10 min each time in PBST buffer followed by incubation with horseradish peroxidase (HRP)-conjugated antibodies at 25℃ for 1–2 h. Different protein bands were then colored using ECL Western Blotting Substrate (Millipore, Billerica, MA, USA). Quantification of protein expression levels was performed by Image J software.

### Immunoprecipitation (IP)

Protein A/G PLUS-Agarose (Santa Cruz Biotechnology, Santa Cruz, CA, USA) was used to pre-purify proteins. Tissue lysates (500 µg) were mixed with anti-CD36 (Proteintech, Rosemont, IL, USA) or normal rabbit IgG (A7016, Beyotime, Haimen, China) and put in the incubator shaker (60 rpm) for one night at 4 °C. Protein-antibody complexes were captured using 20 µl of Protein A/G PLUS-Agarose in the incubator shaker (60 rpm) for another night at 4 °C. They were washed with precooled PBS containing 1 mM phenylmethylsulfonyl fluoride the next day. Equal amounts (5 µL/lane) of proteins were used for western blotting, which could be separated and analyzed by SDS-PAGE.

### Renal function evaluation

Serum creatinine (SCr) levels were assessed utilizing Creatinine (Cr) Assay kit (C011-2-1, Nanjing Jiancheng, Nanjing, China). Blood urea nitrogen (BUN) was quantified utilizing Urea Assay Kit (C013-2-1, Nanjing Jiancheng, Nanjing, China).

### Antibodies

The primary and secondary antibodies used were as follows: anti-CD36 (Santa Cruz Biotechnology; sc-7309, 1:1000 for WB 1:50 for Immunohistochemical (IHC), 1:50 for IF, and 1:100 for IP), anti-dynamin-related protein 1 (DRP1) (Abcam, Cambridge, MA, USA; ab156951, 1:1000 for WB), anti- optic atrophy protein 1 (OPA1)(Abcam, ab119685, 1:1000 for WB), anti-PDGFRβ (Abcam, ab32570, 1:1000 for WB,1:200 for IF), lectin enrichment assay (LCA)-Biotin (Vector Labs, Newark, CA, USA; B-1045, 1:200 for IP), anti-GAPDH (CST, Danvers, MA, USA; 2118, 1:1000 for WB), anti-alpha smooth muscle actin (α-SMA) (Abcam, ab124964, 1:1000 for WB, 1:200 for IHC, 1:200 for IF), anti-Collagen I (Abcam, ab138492, 1:200 for IHC, 1:200 for IF), anti-Fibronectin (Abcam, ab2413, 1:200 for IHC, 1:200 for IF), anti-NG2 (Abcam, ab275024, 1:200 for IF), anti-FUT8 (Biorbyt, Wuhan, China; orb627158, 1:1000 for WB), anti-rabbit IgG, HRP-linked antibody (ZSGB-Bio, Beijing, China; ZB-2301, 1:5000 for WB), and anti-mouse IgG, HRP-linked antibody (ZSGB-BIO, ZB2305, 1:5000 for WB).

### Mitochondrial membrane potential assessment

The mitochondrial membrane potential of pericytes was measured through the JC-1 Assay Kit (C2006, Beyotime). Pericytes were incubated with JC-1 working solution and MitoTracker RED (C1035, Beyotime) for 30 min at 37 °C. Then the liquid was replaced with a fresh culture medium. Confocal laser scanning microscope (SP8, Leica) was used to capture images.

### Statistics

Data are shown as the mean ± SD. The probability (p) values of each experiment were analyzed and plotted in the figure legends with the convenience of GraphPad Prism software (GraphPad Inc., La Jolla, CA, USA). Parametric data’s comparison was performed using two-sample independent t-tests or one-way ANOVA with post-hoc Dunnett or Bonferroni corrections. A p-value < 0.05 was considered statistically significant.

## Results

### The specific change of CD36 in pericytes during AKI-CKD shown in database

**Fig. 1:**
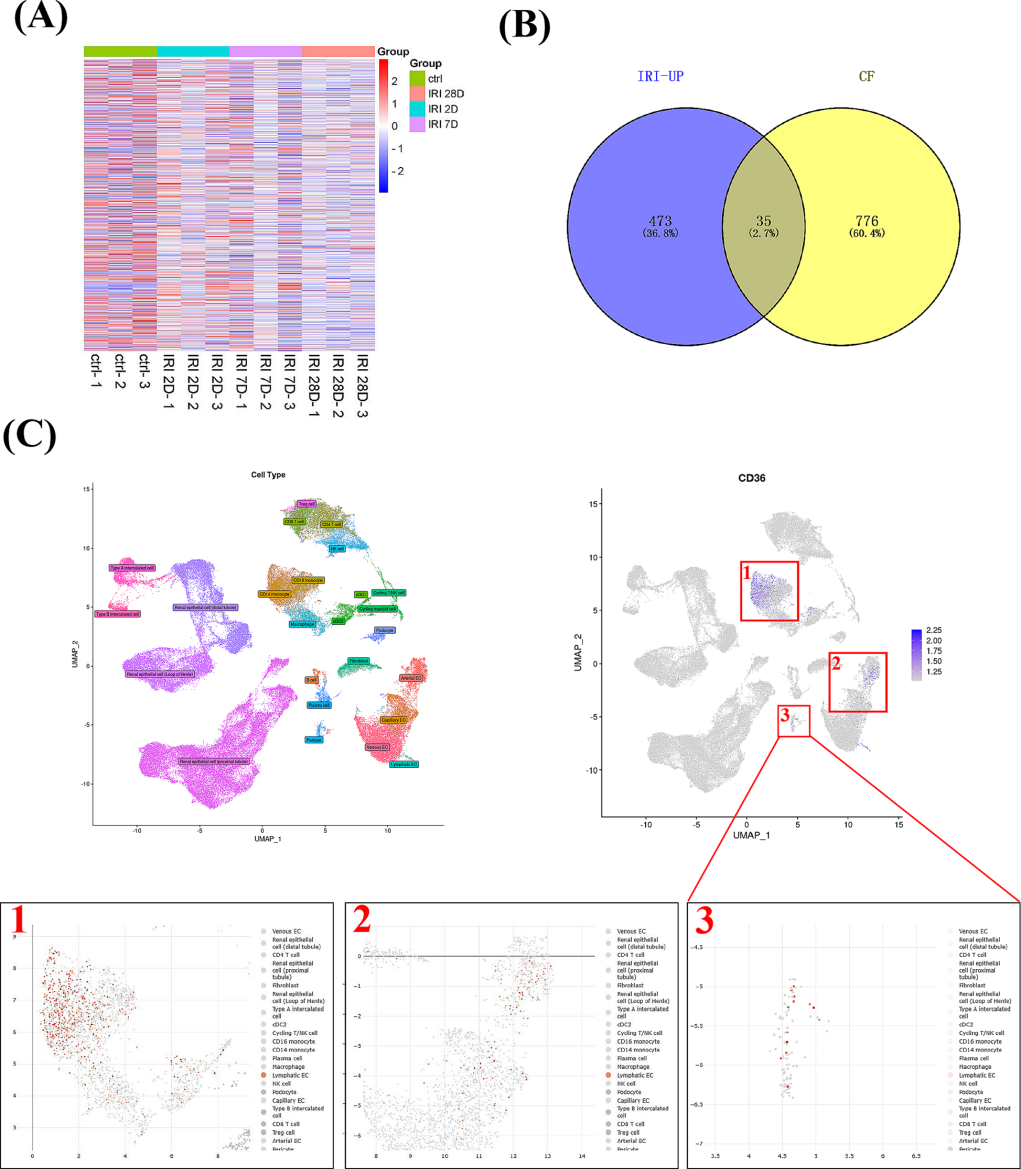
CD36 expression is increased in pericytes during AKI-CKD. (**A**) Heat map of gene expression profile data using data from mice subjected to bilateral I/R injury in a previous study. (**B**) Differentially expressed genes modified by CF. (**C**) The distribution of CD36 in different cells of kidney tissue by single cell RNA sequencing and enlarged diagrams of CD36 expression in specific cells are shown below. Enlarged Diagram 1 represents monocytes, Enlarged Diagram 2 represents endothelial cells, and Enlarged Diagram 3 represents pericytes. The statistical significance of (**B**) was determined using Fisher’s exact test, *p* < 0.001

To explore the significance of pericyte-to-fibroblast transition in the progression of AKI-CKD, we examined datasets from the gene expression omnibus database (GSE140988) documenting genetic changes in pericytes following renal ischemia-reperfusion injury (Chou et al. 2020). Differential expression analysis identified upregulated genes with p-values < 0.05 (Fig. 1A). Given our previous research indicating the critical role of CF in AKI to CKD progression, we intersected these differentially expressed genes with a database of CF-modified proteins and identified CD36 (Fig. 1B). In the dataset, a model was established by carefully titrating renal ischemia time in mice. CD36 gene expression was increased on 2D, recovered by 7D, and then gradually increased up to day 30 post-long I/R injury, respectively. Subsequent single-cell sequencing data from the deeply integrated human single-cell omics database revealed that CD36 is predominantly expressed in monocytes, endothelial cells, and pericytes in the kidney (Fig. 1C). These findings suggest that CD36 might play an essential role in pericyte transdifferentiation and is potentially regulated by CF.

### High expression of CD36 in pericytes during the progression of AKI-CKD

**Fig. 2:**
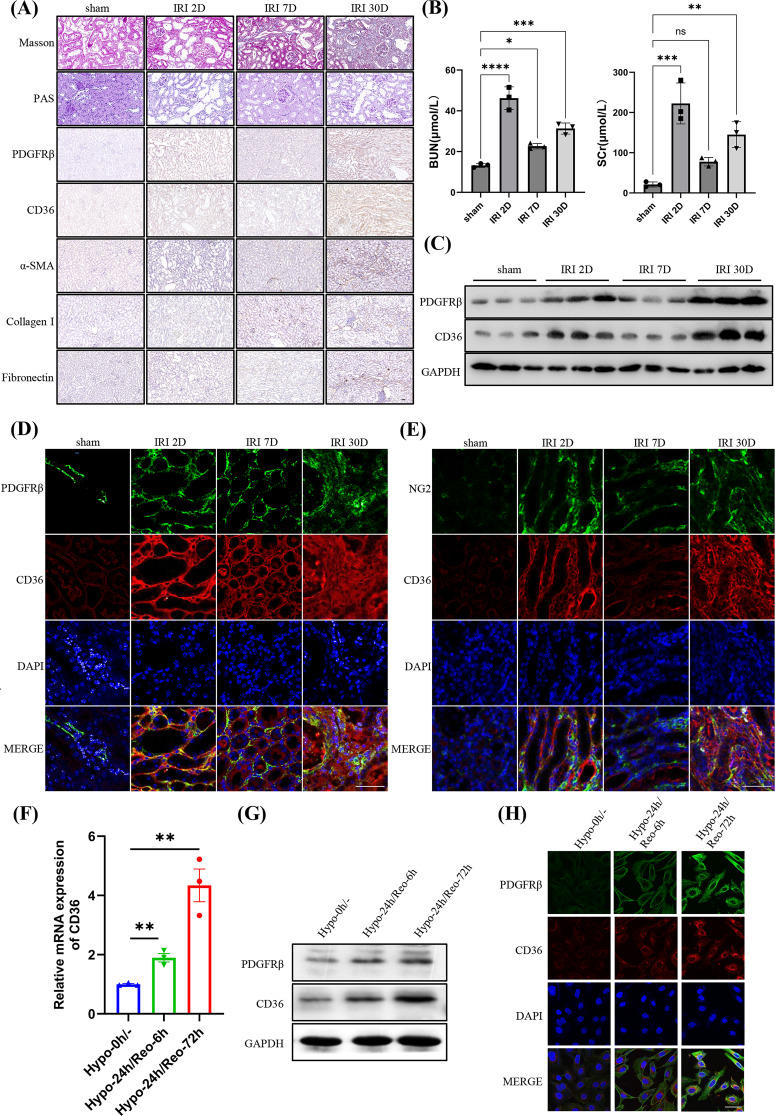
High expression of CD36 in IRI mouse model and pericytes following H/R. (**A**) Representative morphological changes (PAS and Masson staining) and immunohistochemistry images of PDGFRβ, CD36, α-SMA, Collagen I and Fibronectin levels during AKI-CKD (at 2, 7, and 30 days) in wild-type mice (bar = 50 μm, *n* = 3). (**B**) The levels of SCr and BUN in I/R induced mice renal injury (*n* = 3). (**C**) Representative western blotting images of CD36 and PDGFRβ in kidney tissues of the AKI-CKD mouse model (*n* = 3). (**D**) Representative images of CD36 (red) and PDGFRβ (green) by immunofluorescence staining (bar = 50 μm). (**E**) Representative images of CD36 (red) and NG2 (green) by immunofluorescence staining (bar = 50 μm). Primary pericytes of mice were extracted and subjected to H/R (24 h of hypoxia followed by 6–72 h reoxygenation). (**F**) Real-time qPCR detection of CD36 expression in pericytes (Hypo-0 h/-, Hypo-24 h/Reo-6 h and Hypo-24 h/Reo-72 h). CD36 expression levels were normalized to that of GAPDH and presented as relative expression levels (*n* = 3). (**G**) Representative western blotting images of CD36 and PDGFRβ in pericytes. (**H**) Representative immunofluorescence staining images of CD36 (red) and PDGFRβ (green) (bar = 50 μm). ns, no difference; *p < 0 0.05; **p < 0.01; ***p < 0.001; ****p < 0.0001, one-way ANOVA was used to determine statistical significance. Data were presented as mean ± SD

Although it has been extensively studied in the context of kidney disease, the regulatory mechanisms of CD36 in pericytes during AKI-CKD progression remain unclear. First, we established a mouse ischemia-reperfusion injury model and identified AKI-CKD using Masson’s Trichrome, PAS, fibrosis marker α-SMA, Collagen I, Fibronectin staining, and BUN, SCr levels confirming the successful establishment of the model, as previously reported (Li et al. 2023) (Fig. 2A-B; Supplemental Fig. A). Our results also revealed a positive correlation between the expression level of PDGFRβ, which serves as a marker of pericellular activation, and the progression of AKI-CKD. IHC analysis showed that pericytes proliferated and transformed into fibroblasts during the progression of AKI-CKD (Fig. 2A; Supplemental Fig. A). Next, to explore the expression of CD36 in pericytes during AKI-CKD progression, we simultaneously detected the expression of CD36 and found that its level was consistent with the progression of AKI-CKD, showing a sharp increase at IRI day 2 (2D), a slight decrease at 7D, and a subsequent increase at 30D, following the development of AKI–CKD (Fig. 2C; Supplemental Fig. B). Additionally, IF revealed the co-localization of CD36/PDGFRβ and CD46/NG2, NG2 is also a marker of pericytes (Fig. 2D, E; Supplemental Fig. C, D). Furthermore, we extracted primary renal pericytes (Supplemental Fig. E-G), which were used to construct hypoxic reoxygenation (H/R) models in vitro, as previously reported (Li et al. 2023). We found that with prolonged reoxygenation time, the mRNA and protein expression of CD36 in pericytes gradually increased along with the degree of differentiation at 6 h and 72 h after hypoxia-reoxygenation (Fig. 2F, G; Supplemental Fig. H). Confocal microscopy showed the co-location of CD36 and PDGFRβ, and the results of quantitative immunofluorescence analysis also showed the expression of CD36 and PDGFRβ increased along with the reoxygenation time (Fig. 2H; Supplemental Fig. I).

### CD36 knockdown weakens the myofibroblast transformation of Pericyte induced in a hypoxia-reoxygenation cell model

**Fig. 3:**
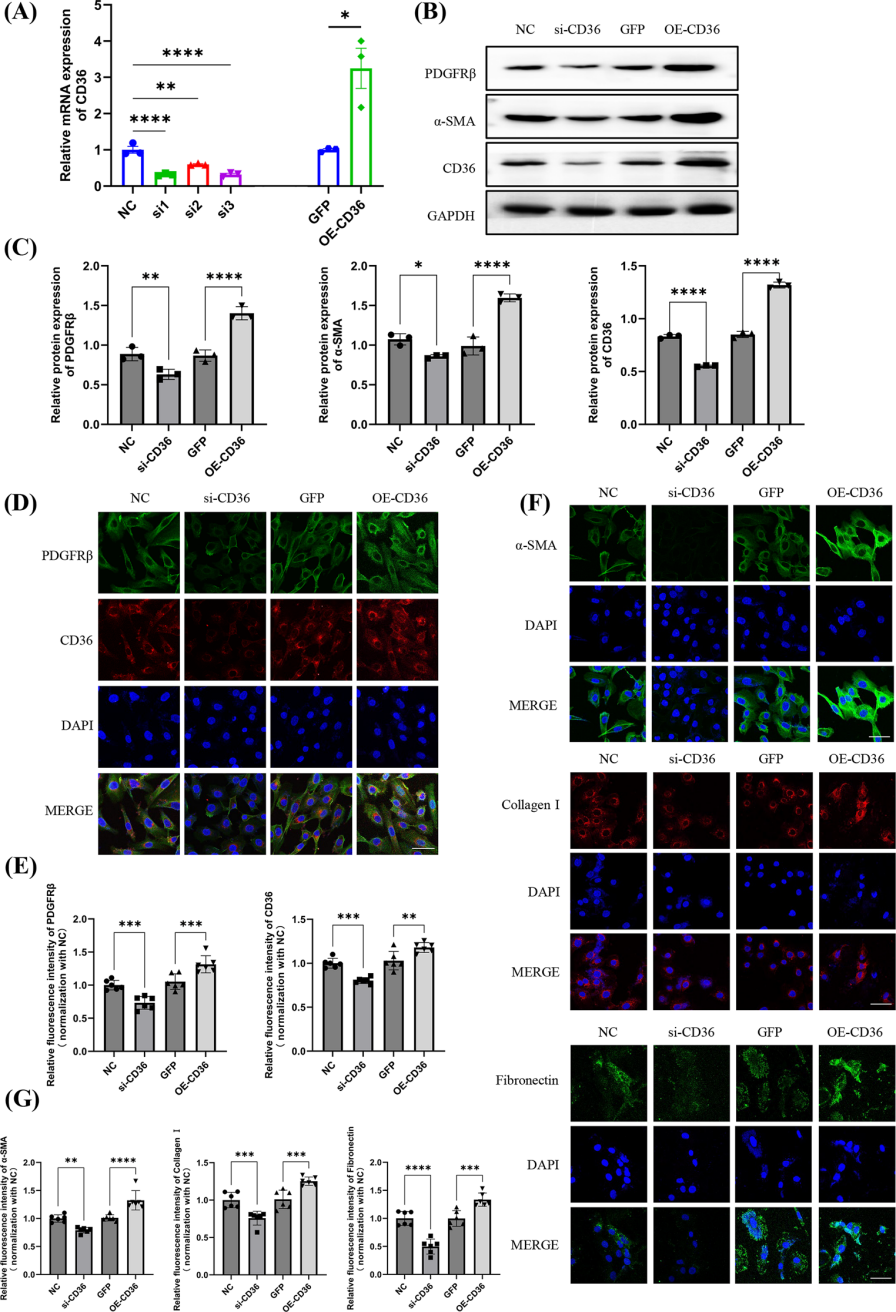
CD36 exacerbates H/R-induced pericyte-myoblast transition in pericytes. Primary pericytes of mice were extracted and subjected to H/R (24 h of hypoxia followed by 72 h reoxygenation) and then transfected with CD36 siRNA and overexpression plasmids. (**A**) Real-time qPCR detection of CD36 gene expression in pericytes. CD36 expression levels were normalized to that of GAPDH and presented as relative expression levels (*n* = 3). (**B**) Representative western blotting data of CD36, PDGFRβ, and α-SMA in pericytes upon knockdown or overexpression of CD36 under H/R condition. (**C**) Quantification of CD36, PDGFRβ, and α-SMA levels (*n* = 3). (**D**) Immunofluorescent staining of CD36 and PDGFRβ (bar = 50 μm). (**E**) Quantify the average fluorescence intensity (*n* = 3). In each biological replicate, two fields were assessed as technical replicates. (**F**) Representative images of immunofluorescent staining for α-SMA, Collagen I and Fibronectin in pericytes (bar = 50 μm). (**G**) Quantify the average fluorescence intensity (*n* = 3). In each biological replicate, two fields were assessed as technical replicates. ns, no difference; *p < 0 0.05; **p < 0.01; ***p < 0.001; ****p < 0.0001, one-way ANOVA was used to determine statistical significance. Data were presented as mean ± SD

To investigate the regulatory mechanism of CD36 in the pericyte model of AKI-CKD, we knocked down its expression using specific short interfering RNAs (siRNAs) or overexpressed CD36 by transfecting OE plasmids into pericytes. The results showed mRNA level of CD36 was decreased by transfecting siRNA 1, siRNA 2 and siRNA 3 (Fig. 3A). We selected CD36 siRNA 3 for subsequent experiments because of its highest knock-down efficiency. We also found that overexpression of CD36 resulted in an increase in both mRNA and protein levels of CD36 in pericytes subjected to 24 h of hypoxia followed by 72 h of reoxygenation. Subsequently, we found that CD36 knockdown significantly reversed the increased expression of α-SMA and PDGFRβ mediated by H/R. Conversely, overexpression of CD36 increased the H/R induced upregulation of α-smooth muscle actin and PDGFRβ (Fig. 3B, C). Similar findings, including Collagen I and Fibronectin, were noted in immunofluorescence assays (Fig. 3D-G). These findings preliminarily suggested that CD36 is closely related to pericyte myofibroblast transformation in the progression of AKI-CKD.

### CD36 contributes to the mitochondrial-dependent apoptosis pathway in pericytes under hypoxia

**Fig. 4:**
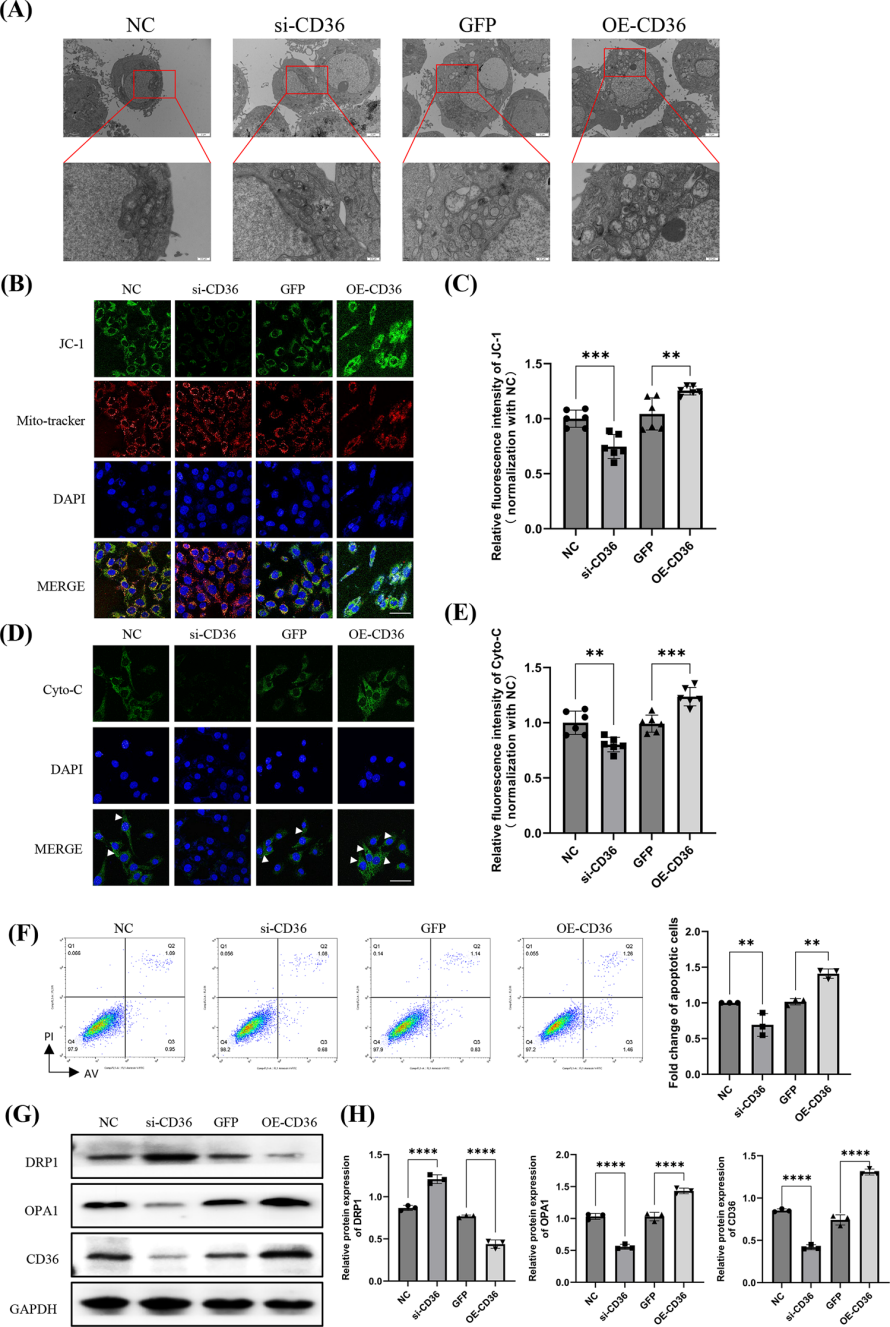
CD36 induced pericyte apoptosis via mitochondria dysfunction under H/R conditions. Primary pericytes of mice were extracted and subjected to H/R (24 h of hypoxia followed by 72 h reoxygenation) and then transfected with CD36 siRNA and overexpression plasmids. (**A**) Representative electron micrograph images. Enlarged images to show the damage are included. (**B**) JC-1 staining to determine the change in the mitochondrial potential (bar = 50 μm). (**C**) Quantify the average fluorescence intensity (*n* = 3). In each biological replicate, two fields were assessed as technical replicates. (**D**) Cytochrome C (Cyto-C) was observed moving from the inter-mitochondrial space into the cytosol using immunofluorescence imaging analysis (bar = 50 μm). (**E**) Quantify the average fluorescence intensity (*n* = 3). In each biological replicate, two fields were assessed as technical replicates. (**F**) Pericyte apoptosis was then determined by FACS analysis (*n* = 3). (**G**) Representative western blotting data for DRP1, OPA1, and CD36 in peri-cytes upon knockdown or overexpression of CD36 under H/R conditions. (**H**) Quantification of DRP1, OPA1 and CD36 levels (*n* = 3). ns, no difference; *p < 0 0.05; **p < 0.01; ***p < 0.001; ****p < 0.0001, one-way ANOVA was used to determine statistical significance. Data were presented as mean ± SD

It has been reported that CD36 is associated with mitochondrial dysfunction (Niu et al. 2023). To further identify the underlying molecular mechanisms by which CD36 regulates pericyte behavior under H/R conditions, we analyzed the apoptosis pathway induced by mitochondrial dysfunction. TEM analysis revealed that H/R led to the formation of large spherical mitochondria, which contain the outer mitochondrial membrane, inner mitochondrial membrane, and the mitochondrial matrix, but appear fewer cristae compared with normal mitochondria. Meanwhile, the mitochondrial swelling and membrane rupture induced by H/R in pericytes were inhibited by knocking down CD36, but became worse when CD36 was overexpressed (Fig. 4A). To probe the effect of hypoxia on mitochondrial morphology deeply, we employed JC-1 staining to identify alterations in the mitochondrial membrane potential. The results showed that knocking down CD36 suppressed the alterations in mitochondrial membrane potential in injured cells, which was reversed by CD36 overexpression (Fig. 4B, C). In addition, knocking down CD36 reduced hypoxia-induced increases in cytochrome C release, which release was increased by overexpressing CD36 in pericytes under H/R (Fig. 4D, E). Furthermore, knocking down CD36 inhibited the number of H/R-induced apoptotic cells and the levels of markers expression of mitochondria damage (including DRP1 and OPA1, as detected using flow cytometry and WB (Fig. 4F-H). These results preliminarily demonstrated that CD36 can regulate mitochondrial damage-induced apoptotic pathways in pericytes under H/R conditions.

### CD36 undergoes core fucosylation modification in pericytes

**Fig. 5:**
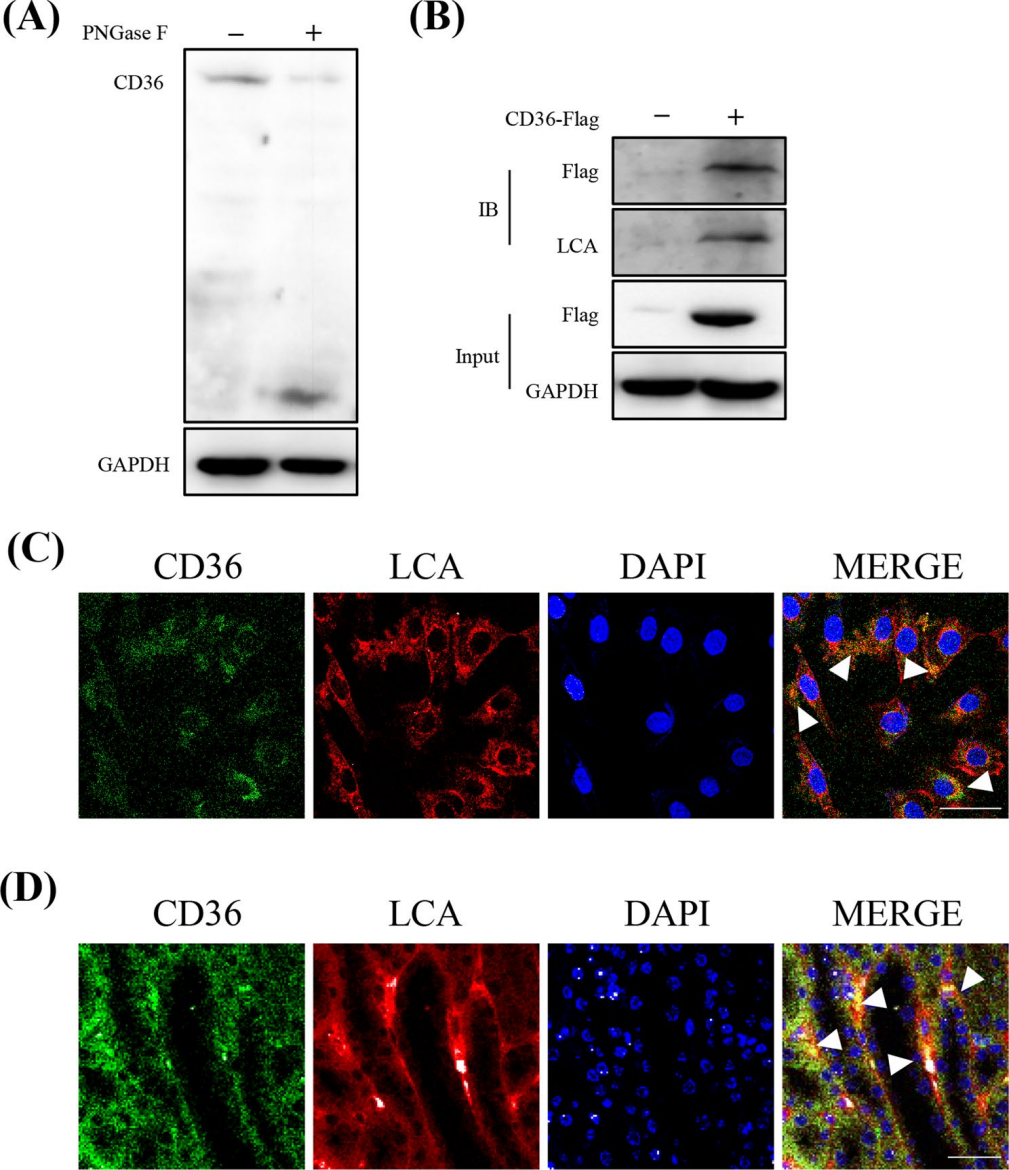
CD36 is N-glycosylated and core fucosylated in pericytes under H/R conditions. (**A**) Western blotting of CD36 was treated with or without PNGase F at 37 °C for 3 h. (**B**) LCA blot of FLAG-CD36 immunoprecipitated using anti-FLAG beads. (**C**) Immunofluorescent staining of LCA and CD36 in pericytes (under H/R conditions) (bar = 50 μm). (**D**) Immunofluorescent staining of LCA and CD36 in kidney tissues (AKI-CKD mouse model) (bar = 50 μm)

The above results indicated that CD36 plays a significant role in pericyte injury under H/R. Therefore, we next aimed to explore how CD36 is activated. CD36 serves as a multifunctional glycoprotein, functioning as a receptor for a diverse array of ligands, and it is mainly expressed on the cell membrane (Wang and Li 2019); therefore, we detected the level of CD36 and CF on the cell membrane. First, we confirmed the existence of N-glycosylation and CF on CD36 in pericytes under H/R conditions. We analyzed the western blotting of CD36 in pericytes after the treatment with PNGase F, which specifically cleaves whole N-glycans. We found that the western blotting of CD36 was shifted from 88 kDa to approximately 35 kDa, suggesting the presence of extensive N-glycans on CD36 in pericytes (Fig. 5A). To further clarify that CD36 was modified by CF, we constructed a CD36-FLAG plasmid, and exogenous detection again confirmed that CD36 was modified by CF. We overexpressed CD36-FLAG in pericytes and pulled it down using FLAG-labeled beads. The co-IP proteins were detected by western blotting with a lectin enrichment assay. The results demonstrated that CF existed on the CD36 protein (Fig. 5B). Consistently, co-localization of CD36 and CF was detected in pericytes under H/R condition and in the kidney tissues of the AKI-CKD mouse model (Fig. 5C, D).

### FUT8 mediates core fucosylation of CD36 in pericytes

**Fig. 6:**
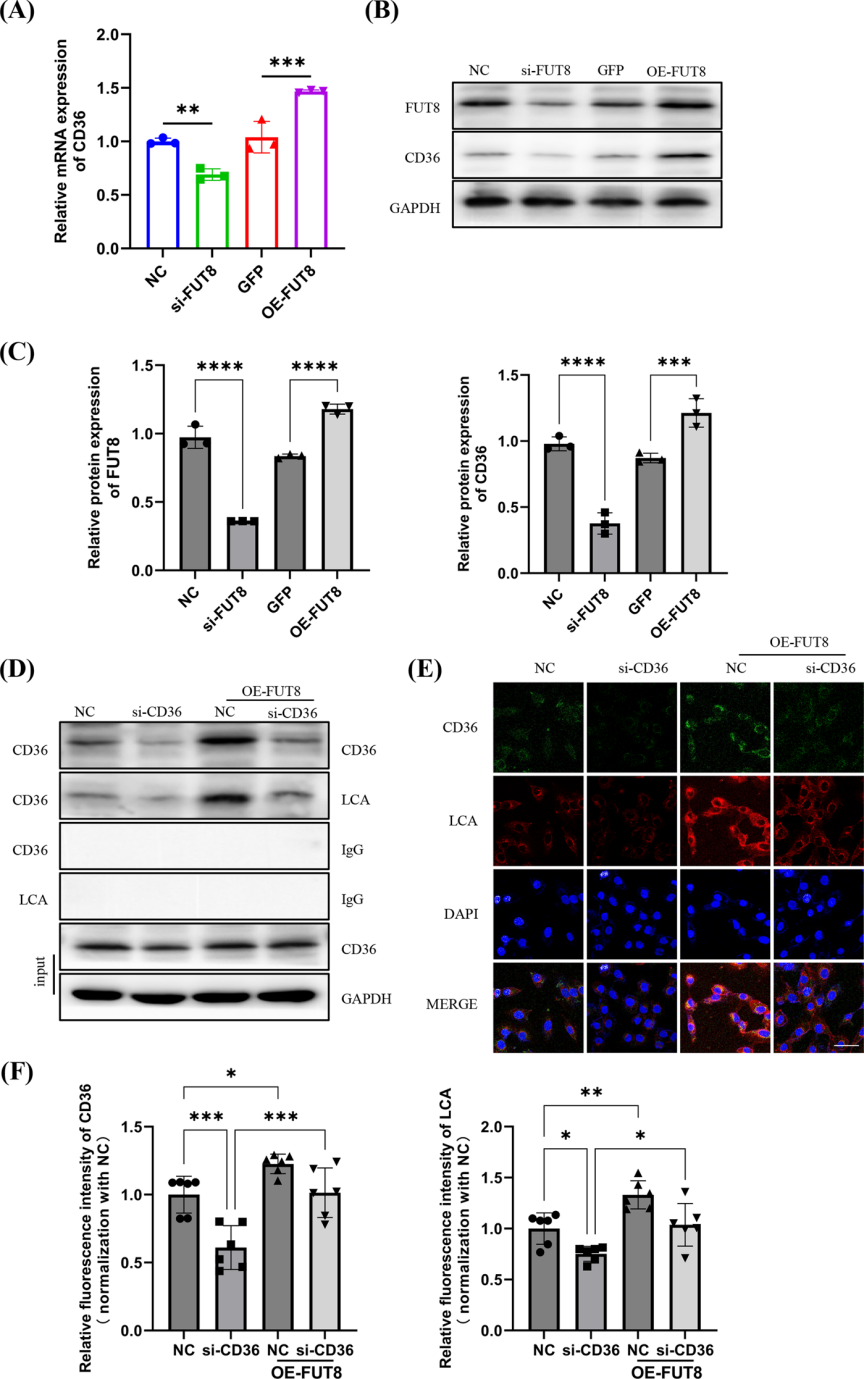
FUT8 mediates the core fucosylation of CD36 in pericytes. (**A**) Real-time qPCR of CD36 in pericytes upon knockdown or overexpression of FUT8 under H/R conditions (*n* = 3). (**B**) Representative western blotting data of CD36 and FUT8 in pericytes upon knock-down or overexpression of FUT8 under H/R conditions. (**C**) Quantification of FUT8 and CD36 levels (*n* = 3). (**D**) LCA blot of FLAG-CD36 immunoprecipitated using anti-FLAG beads upon knockdown of CD36 and/or overexpression of FUT8 under H/R conditions. (**E**) Immunofluorescent staining of LCA and CD36 in pericytes upon knockdown of CD36 and/or overexpression of FUT8 under H/R conditions (bar = 50 μm). (**F**) Quantify the average fluorescence intensity (*n* = 3). In each biological replicate, two fields were assessed as technical replicates. ns, no difference; *p < 0 0.05; **p < 0.01; ***p < 0.001; ****p < 0.0001, one-way ANOVA was used to determine statistical significance. Data were presented as mean ± SD

As the only enzyme catalyzing CF, FUT8 was speculated to have a key role in regulating CD36 in pericytes. Accordingly, we transfected pericytes with FUT8-specific siRNAs or overexpression plasmids. At 48 h post-transfection, CD36 protein and mRNA levels were assessed, and we found that CD36 expression was consistently decreased after FUT8 knockdown, but was increased by FUT8 overexpression (Fig. 6A-C). Furthermore, we performed a rescue experiment by knocking down CD36 and then transfecting with the FUT8 overexpression plasmid to detect its effects on CD36 protein levels and its CF levels. An immunoprecipitation assay showed that CF of CD36 was markedly reduced by CD36 knockdown, but increased by FUT8 overexpression (Fig. 6D). Moreover we found that both CD36 protein levels and its CF modification levels were suppressed following CD36 knockdown (Fig. 6D). However, overexpression of FUT8 was able to partially restore both the expression and the CF modification levels of CD36 (Fig. 6E, F). The results collectively indicated a regulatory effect of CD36 and its CF modification by FUT8 in pericytes under H/R.

### FUT8 regulates the CD36-mediated mitochondrial apoptosis pathway to induce pericyte myofibroblast transformation

**Fig. 7:**
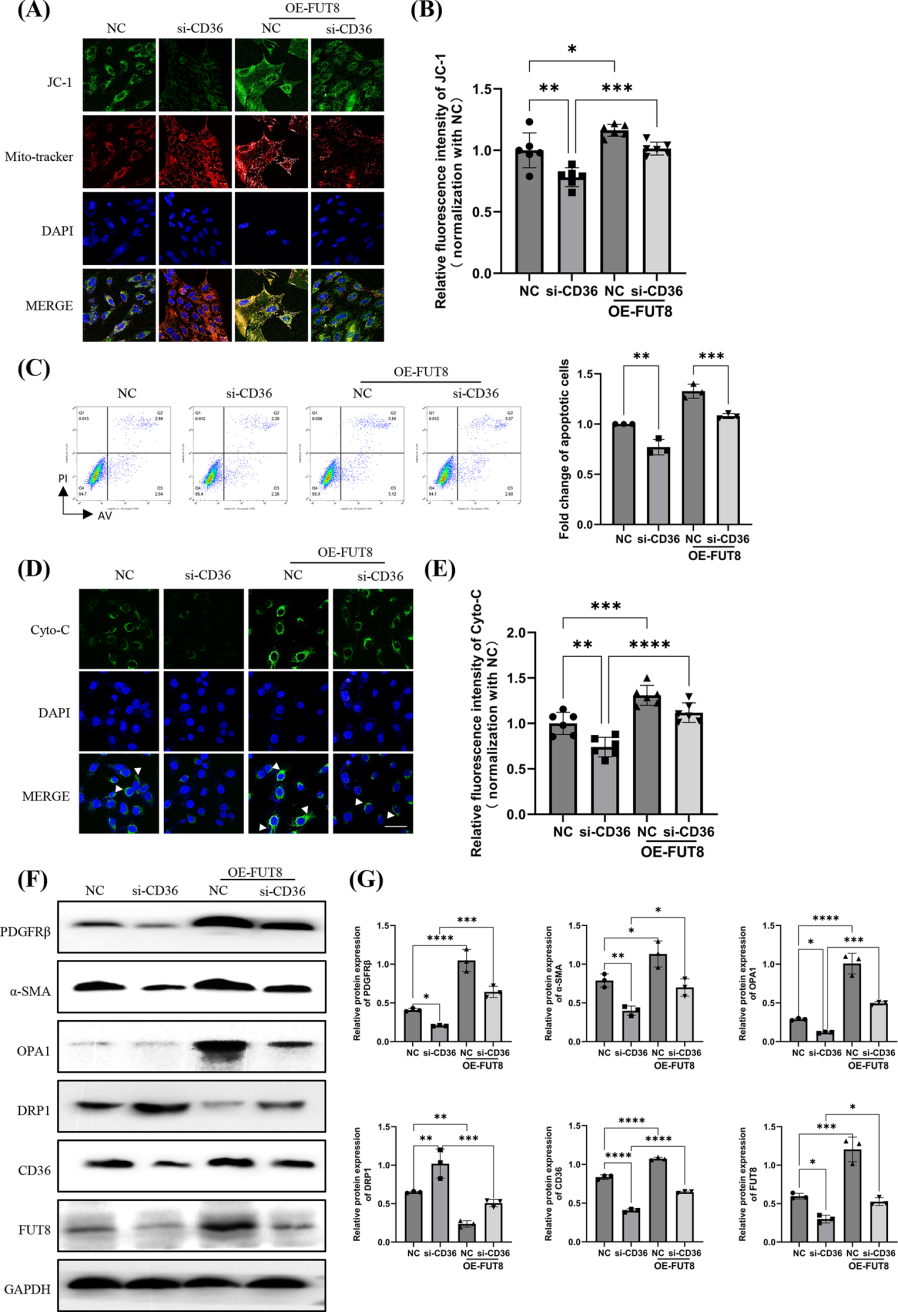
The FUT8-CD36/CF axis regulates the mitochondrial apoptosis pathway and promotes pericyte trans-differentiation. Pericytes were transfected by the empty vector and FUT8 overexpression plasmid, and the siRNA against CD36 was transfected into the cells. (**A**) JC-1 staining to determine the change in the mitochondrial potential (bar = 50 μm). (**B**) Quantification of the mean fluorescence intensity (*n* = 3). In each biological replicate, two fields were assessed as technical replicates. (**C**) Pericyte apoptosis was determined using FACS analysis (*n* = 3). (**D**) Cytochrome C (cyto-c) was observed moving from the inter-mitochondrial space into the cytosol using immunofluorescence imaging analysis (bar = 50 μm). (**E**) Quantify the average fluorescence intensity (*n* = 3). In each biological replicate, two fields were assessed as technical replicates. (**F**) Representative western blotting of the protein levels of CD36, DRP1, OPA1, FUT8, α-SMA, and PDGFRβ in pericytes upon knockdown CD36 and/or overexpression of FUT8 under H/R conditions. (**G**) Quantification of CD36, DRP1, OPA1, FUT8, α-SMA, and PDGFRβ levels (*n* = 3). ns, no difference; *p < 0 0.05; **p < 0.01; ***p < 0.001; ****p < 0.0001, one-way ANOVA was used to determine statistical significance. Data were presented as mean ± SD

Considering the combined promising myofibroblast transformation effect of CD36 and FUT8 in kidney fibrosis, we further investigated the potential relationship between CD36 and FUT8 expression in pericytes(Kuppe et al. 2021; Shen et al. 2013; Okamura et al. 2009). However, whether FUT8 regulates the mitochondrial apoptosis pathway through CD36 remains unknown. Therefore, pericytes were induced to overexpress FUT8 after CD36 silencing. At 48 h post-transfection the mitochondrial membrane potential, cell apoptosis and pericyte myofibroblast transformation were assayed. The results indicated that overexpression of FUT8 exacerbated the changes in mitochondrial membrane potential induced by CD36 knockdown in pericytes (Fig. 7A, B). Flow cytometry experiments also showed that FUT8 overexpression further increased the reduction in apoptosis induced by CD36 knockdown in pericytes (Fig. 7C), as evidenced by cytochrome C release (Fig. 7D, E) and the expression of DRP1 and OPA1 (Fig. 7F, G). Additionally, FUT8 overexpression restored the suppressed expression of α-SMA induced by CD36 knockdown (Fig. 7F, G). These findings demonstrated that targeting CD36 via FUT8 could promote mitochondrial injury, induce apoptosis, and thereby facilitate pericyte myofibroblast transformation.

## Discussion

This study focused on the role of renal pericytes in the AKI to CKD transition, investigating their transformation and mitochondrial apoptosis signaling pathways. Pericytes, a type of cell located around capillaries and small blood vessels (Li and Fan 2023), are increasingly recognized as key players in renal fibrosis and the progression of kidney diseases (Cao et al. 2024), capillary rarefaction is promoted by the differentiation and detachment of pericytes from peritubular capillaries, which is considered an important mechanism that exacerbates tissue hypoxia, leading to the progression of CKD (Shih et al. 2018; Tanaka et al. 2023). However, there is limited research on the role of pericytes in AKI-CKD, and it is important to understand the mechanisms behind the pericyte-myofibroblast transition. This study highlighted the pivotal role of renal pericytes in the progression from AKI to CKD in vivo and in vitro. By understanding the mechanisms driving pericyte transformation, we can identify potential targets for therapeutic intervention to halt or reverse the development of CKD. Herein, Chou et al. reported that following AKI to CKD progression, renal pericytes exhibited substantially increased expression of markers associated with myofibroblast transformation, such as α-SMA and PDGFRβ (Chen et al. 2011; Wu et al. 2013; Chang et al. 2012). In contrast to the fibrotic role of myofibroblasts in CKD, AKI leads to the transient activation of myofibroblasts, which includes the injured tubular epithelium, and promotes tubular regeneration (Venkatachalam et al. 2015). However, as myofibroblasts undergo apoptosis, the kidney experiences a brief recovery phase, but does not return to baseline levels (Maremonti et al. 2022). Pericytes proliferate as an important source of myofibroblasts during the AKI-CKD transition and detach as the damage progresses, resulting in the destabilization of the microvascular system, which ultimately leads to the occurrence of interstitial fibrosis(Liu 2011). Due to renal damage, pericytes proliferate during the initial phase of IRI, followed by a brief pause of pericytes proliferation as facilitated renal tissue repair. However, continuous stimulation disrupted the normal repair process, leading to an initiation of inflammation-fibrosis transition, this maladaptive repair process maintained the abnormal activation of pericytes, which disrupted the stability of blood vessel walls, thereby promoting the development of renal fibrosis. This may account for the observed results that PDGFRβ expression following IRI exhibited an increase on day 2, a decrease on day 7, and a subsequent rise again on day 30. While further research is needed to delineate the significance of pericyte-to-myofibroblast transition in AKI, our findings preliminarily highlighted the role of pericyte-to-myofibroblast transition in AKI-to-CKD progression, and emphasized the role of the FUT8-CD36 axis in these processes.

The multifunctional receptor CD36 plays a significant role in various biological processes by activating pathways such as Toll-like receptors (Reed-Geaghan et al. 2009), Na+/K + ATPase (Pillai et al. 2023), the NLR family pyrin domain containing 3 (NLRP)3 inflammasome (Lv et al. 2022), protein kinase C (PKC)-NADPH oxidase (Lin et al. 2012), Src/Lyn/Fyn kinases (Huang et al. 1991; Thorne et al. 2006), mitogen activated protein kinase (MAPK) (Nan et al. 2022), and transforming growth factor beta (TGF-β) signaling (Tao et al. 2022). These activations contribute to lipid accumulation, inflammation, energy reprogramming, apoptosis, and renal fibrosis (Yang et al. 2017; Ma et al. 2024). In this study, bioinformatic analysis of an online database showed that CD36 levels were markedly increased in AKI CKD progression, and CD36 was found to be expressed in pericytes in the single cell sequencing database analysis. We further confirmed that CD36 exhibits dynamic expression changes during CKD progression in a mouse model. Specifically, on day 2 of AKI (the acute phase), CD36 expression increased rapidly. By day 7, as the disease stabilizes, CD36 expression partially recovered. However, as the disease progressed to CKD, CD36 expression increased again. This suggested that CD36 can serve as a predictive marker for AKI-CKD progression. Core fucosylation is necessary for various protein functions. Although CD36 has been reported to be highly glycosylated with N-linked glycans in the literature, it remains obscure whether the sugar moieties contribute to AKI-CKD transition (Agarwal et al. 2024; Hoosdally et al. 2009). Moreover, CD36 was one of the CF-modified proteins screened by proteomics. Further experiments confirmed that CF of CD36 is an important factor in pericyte differentiation, which is associated with AKI-CKD progression and may serve as an adverse indicator of AKI-CKD. Additionally, CD36 is known to undergo N-glycosylation, which has been reported to stimulate neutrophil apoptosis and clearance during cardiac remodeling (DeLeon-Pennell et al. 2016). We also found that CF of CD36 contributed to the induction of the mitochondrial-apoptosis pathway. Notably, our study is the first to report the significant role of CD36 and its CF modification in the mitochondrial apoptosis pathway and the trans-differentiation of renal pericytes during the progression from AKI to CKD.

In our previous studies, we reported that renal tubule epithelial cell-specific knockout of FUT8 has been shown to reduce endothelial cell and pericyte damage via paracrine mechanisms by downregulating the levels of CF modifications in surrounding cells during AKI-CKD progression(Li et al. 2023). Additionally, our team previously reported that FUT8/CF played a crucial role in the pericyte to accelerate the RIF process (using the UUO mouse model) (Wang et al. 2017). However, the regulatory role of FUT8/CF modifications in pericyte injury during the progression from AKI to CKD remains unclear. In this study, we showed that FUT8 is involved in modulating the CF of CD36 in pericytes. FUT8 was crucial for the stability and cell-surface expression of CF-modified CD36, as confirmed by our experiments. In addition, the LCA demonstrated that CF of CD36 is regulated by FUT8. In our findings, it was also evident that the inhibition of pericyte-fibroblast transition was no longer observed following the suppression of FUT8 in CD36-overexpressing cells. We also clarified the important function of FUT8-mediated aberrant CD36 CF in promoting the AKI-CKD progression. The possible involvement of other fucosyltransferases in CD36 glycosylation should be clarified in a further study.

Apoptosis induced by mitochondrial damage is the key molecular mechanism of AKI-CKD progression (Zhang et al. 2021), Yang et al. identified CD36 in purified mitochondria, and suppression of CD36 protein modifications through genetic or pharmacological methods could potentially protect against kidney fibrosis by switching fatty acids from an accumulation to a consumption phenotype (Yang et al. 2017). To date, the mechanisms related to mitochondrial injury that induce cell apoptosis of pericytes with high FUT8-CD36 CF expression are not fully understood. This study builds on previous findings that FUT8/CF modifications play a crucial role in AKI to CKD progression, and reveals a possible mechanism in which FUT8/CF modifications have a critical role in regulating mitochondrial apoptosis pathways. Moreover, the transition of pericytes into myofibroblasts might be partially realized by controlling CD36 expression and the level of its CF modifications. To the best of our knowledge, this is the first report of the involvement of a CF disorder in the mitochondrial apoptosis pathway of pericytes in an AKI-CKD mouse model.

Patients who experience AKI and survive are at greater risk of developing CKD (Kellum et al. 2021). The exact mechanisms and factors influencing this progression are incompletely understood, necessitating further research to identify biomarkers, genetic predispositions, and other risk factors. Early identification of patients at risk of progressing from AKI to CKD could lead to timely interventions that might prevent or slow the progression of kidney disease.

**Fig. 8:**
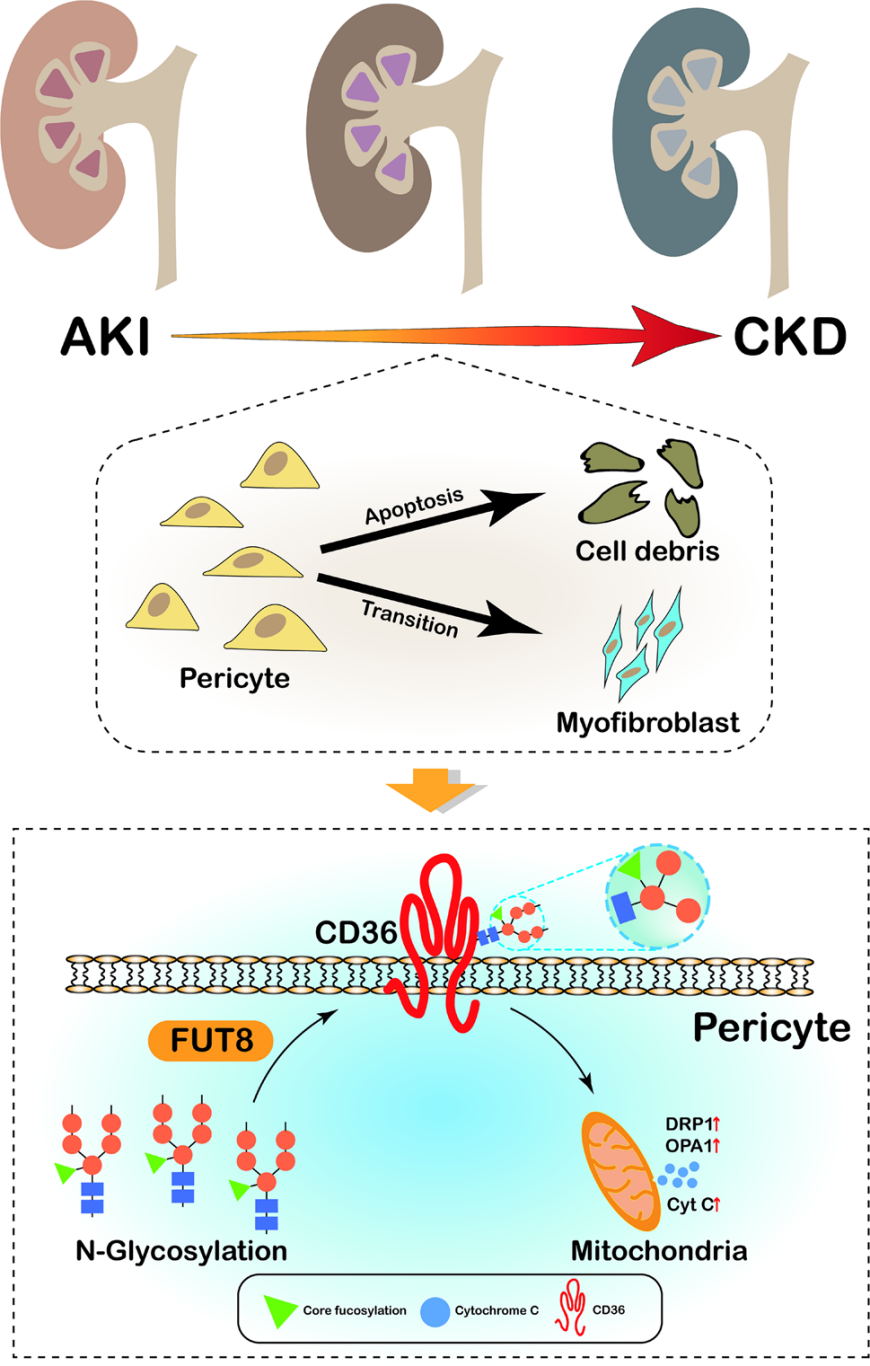
Schematic diagram showing the molecular mechanism of pericyte-fibroblast transition through FUT8-CD36/CF-mitochondrial apoptosis pathway during AKI-CKD

In conclusion, our study extends previous findings, revealing the crucial role of the downstream molecule of FUT8, CD36, in the progression from AKI to CKD. Specifically, CD36 promotes pericyte trans-differentiation by regulating the mitochondrial apoptosis pathway. Additionally, we demonstrated that CD36 is modified by CF, as regulated by FUT8, and upregulated FUT8 expression contributes to the core fucosylation of CD36 and activation of the mitochondrial-dependent apoptosis signaling pathway, which leads to pericyte transition and ultimately aggravates AKI-CKD progression (Fig. 8). This study uncovered the role of FUT8 in regulating CD36 in pericytes, providing insights into the mechanisms underlying pericyte injury and identifying potential therapeutic targets to delay the progression of AKI to CKD, FUT8/CD36-CF axis may be a promising therapeutic strategy for delaying the progression of AKI to CKD in the future.

## Electronic supplementary material

Below is the link to the electronic supplementary material.

Supplementary Material 1

Supplementary Material 2

## Data Availability

All data analyzed in this study are included within the article. Gene expression profile data and differentially expressed genes modified by CF from mice subjected to bilateral I/R injury were extracted from GSE140988 dataset, which was acquired from the GEO database (gene expression omnibus, https://www.ncbi.nlm.nih.gov/geo/) (Chou et al. 2020).). The data of distribution of CD36 in different cells of kidney tissue were achieved from the DISCO database (deeply integrated human single-cell omics database, https://www.immunesinglecell.org) (Li et al. 2022).
