# Supplementary material for: FUT8 upregulates CD36 and its core fucosylation to accelerate pericyte-myofibroblast transition through the mitochondrial-dependent apoptosis pathway during AKI-CKD
Source: Mol Med. 2024 Nov 20;30:222. doi: 10.1186/s10020-024-00994-6 (PMC11577590; doi:10.1186/s10020-024-00994-6)

The full uncropped and unedited versions of Western blots

Figure 2 C

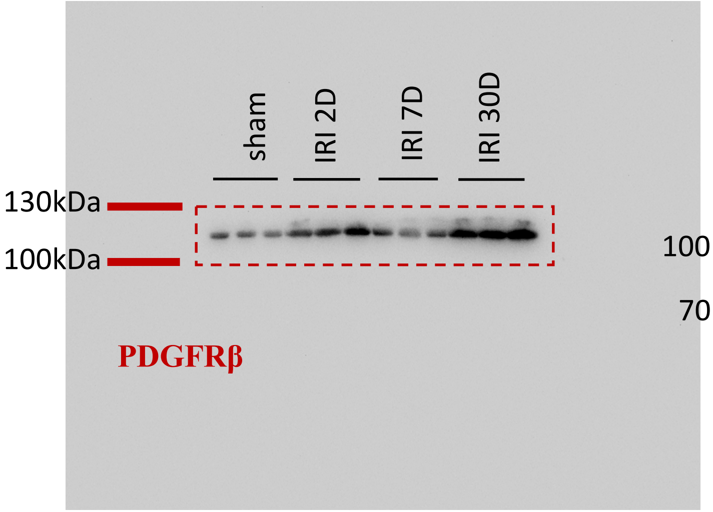

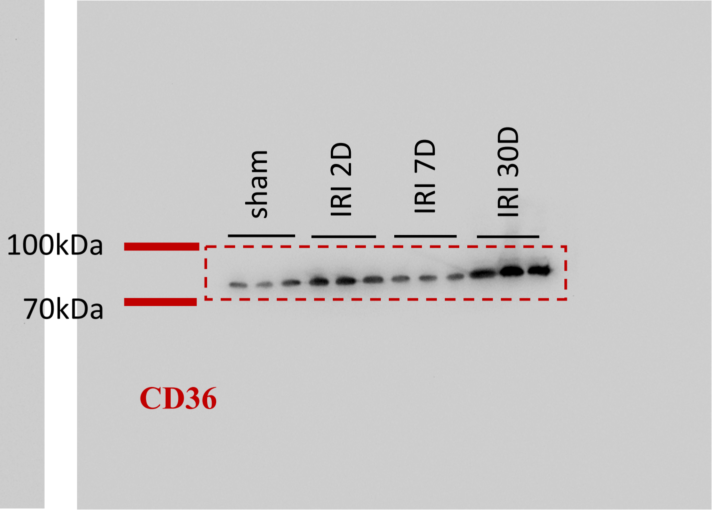

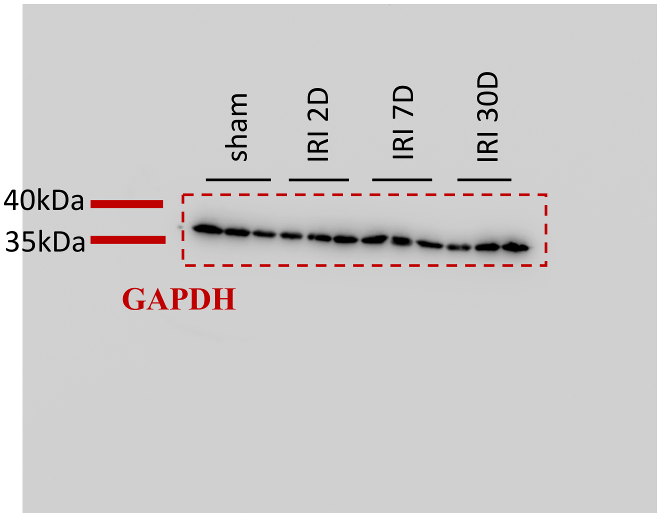

Figure 2 G

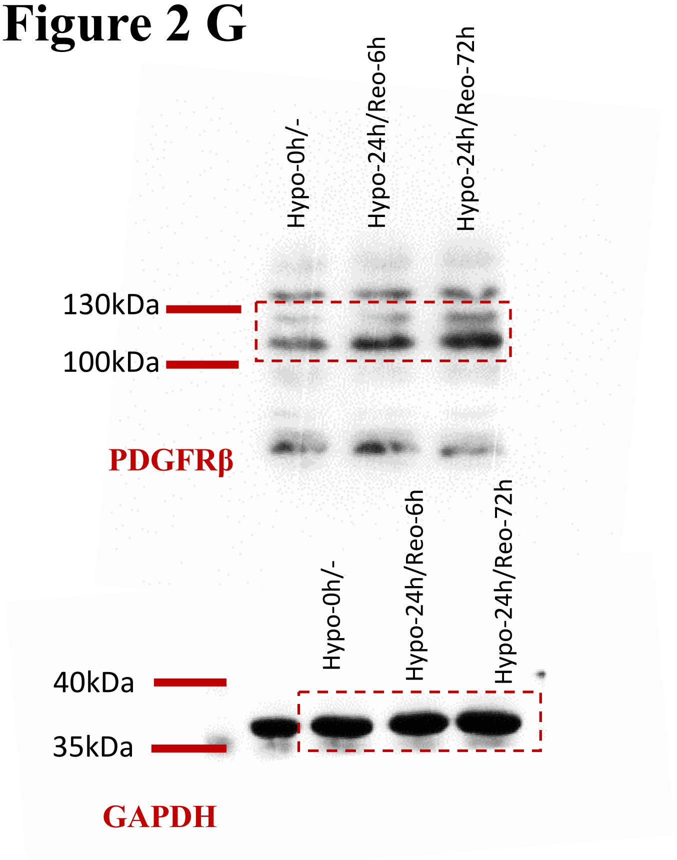

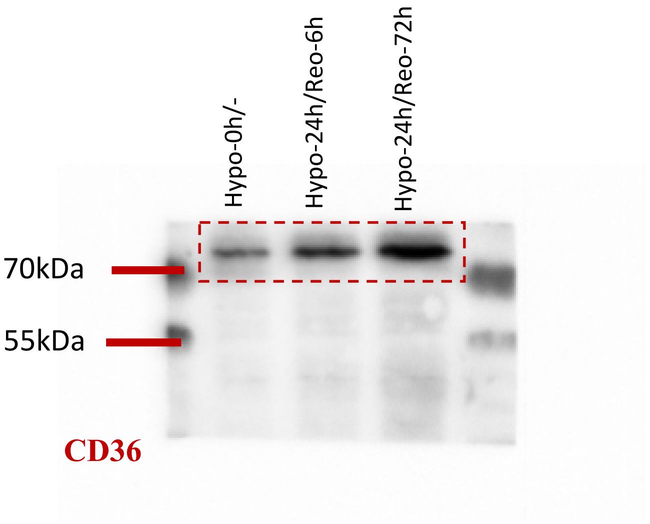

Figure 3 B

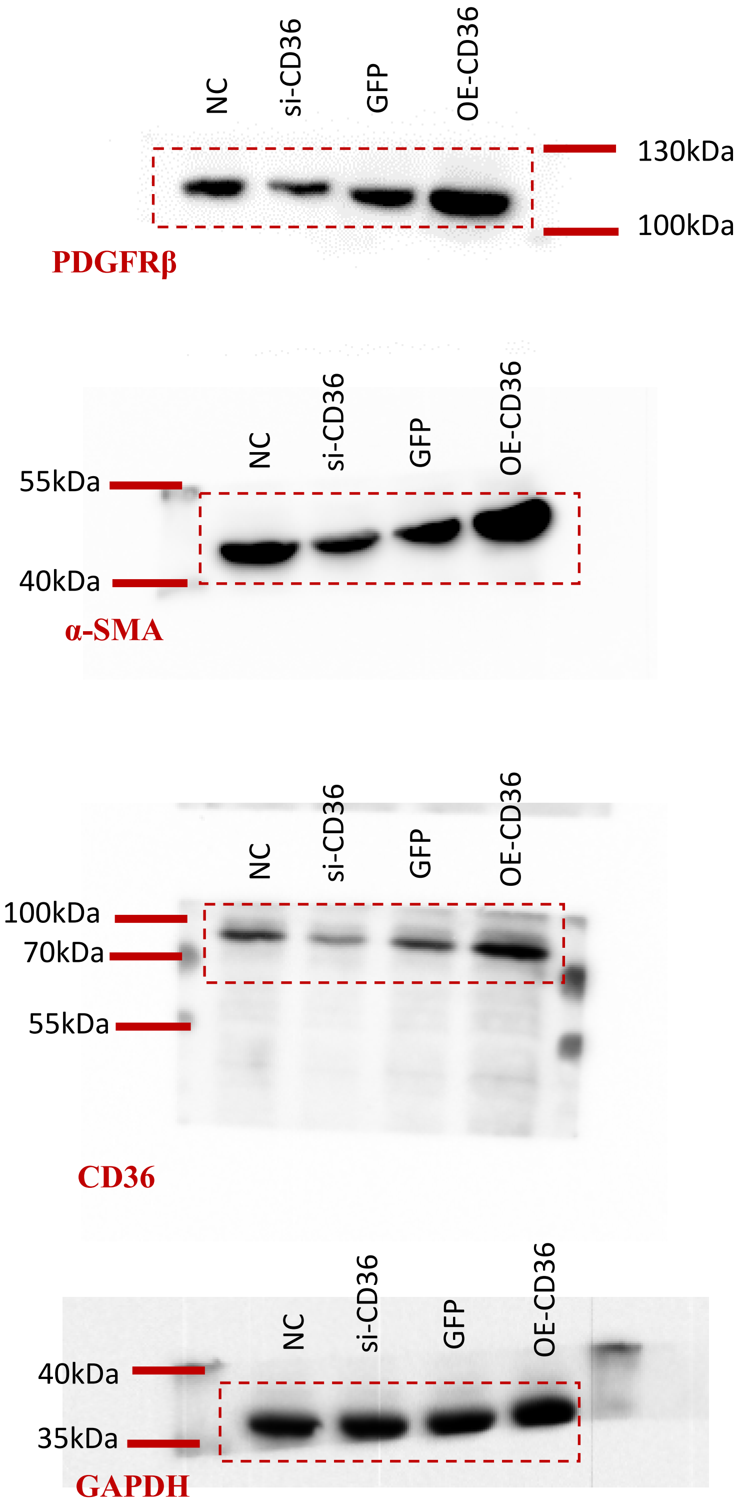

NC
si-CD36
GFP
OE-CD36
130kDa
100kDa
PDGFRβ
NC
si-CD36
GFP
OE-CD36
55kDa
40kDa
α-SMA
NC
si-CD36
GFP
OE-CD36
100kDa
70kDa
55kDa
CD36
NC
si-CD36
GFP
OE-CD36
40kDa
35kDa
GAPDH

Figure 4 G

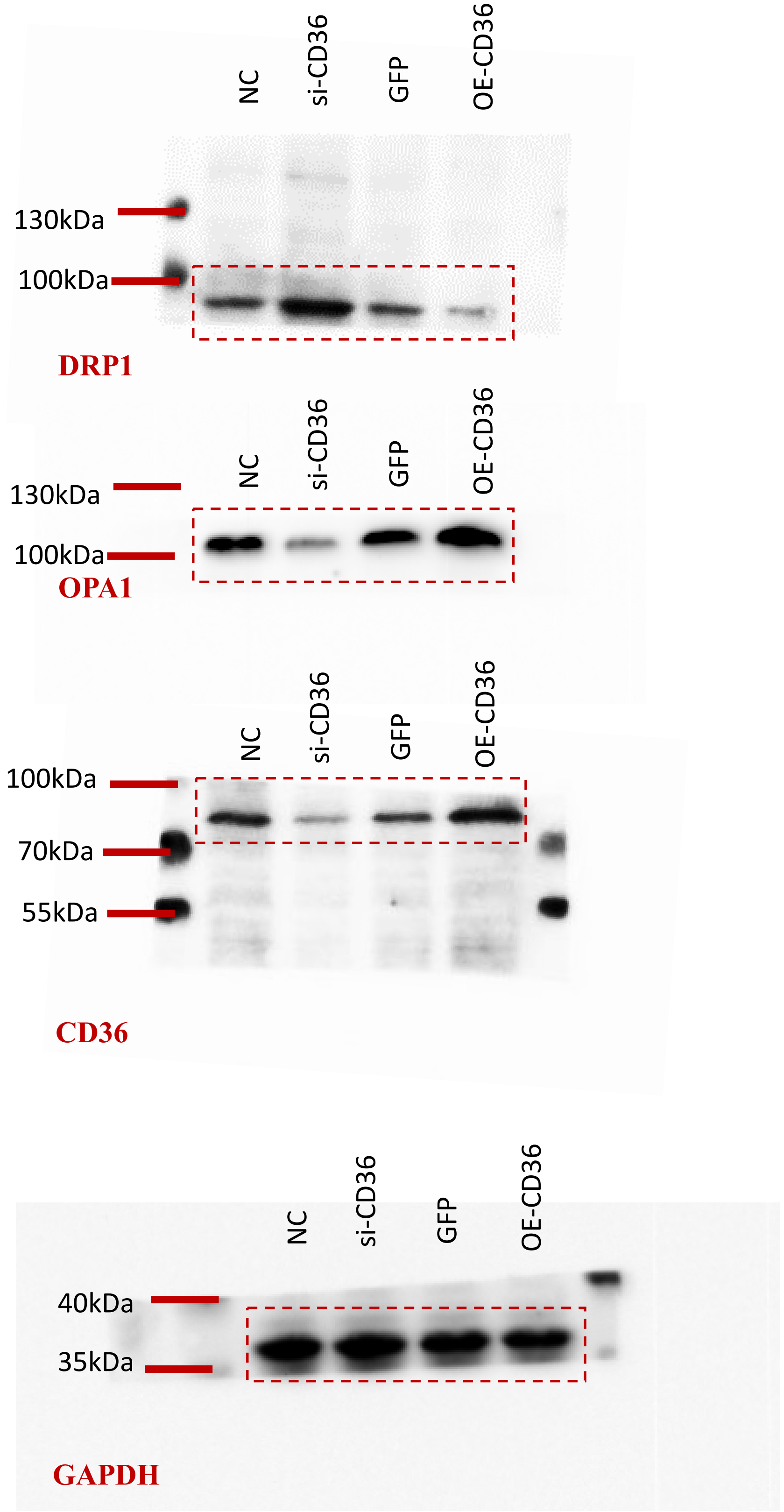
NC
si-CD36
GFP
OE-CD36
130kDa
100kDa
DRP1
NC
si-CD36
GFP
OE-CD36
130kDa
100kDa
OPA1
NC
si-CD36
GFP
OE-CD36
100kDa
70kDa
55kDa
CD36
NC
si-CD36
GFP
OE-CD36
40kDa
35kDa
GAPDH

Figure 5 A

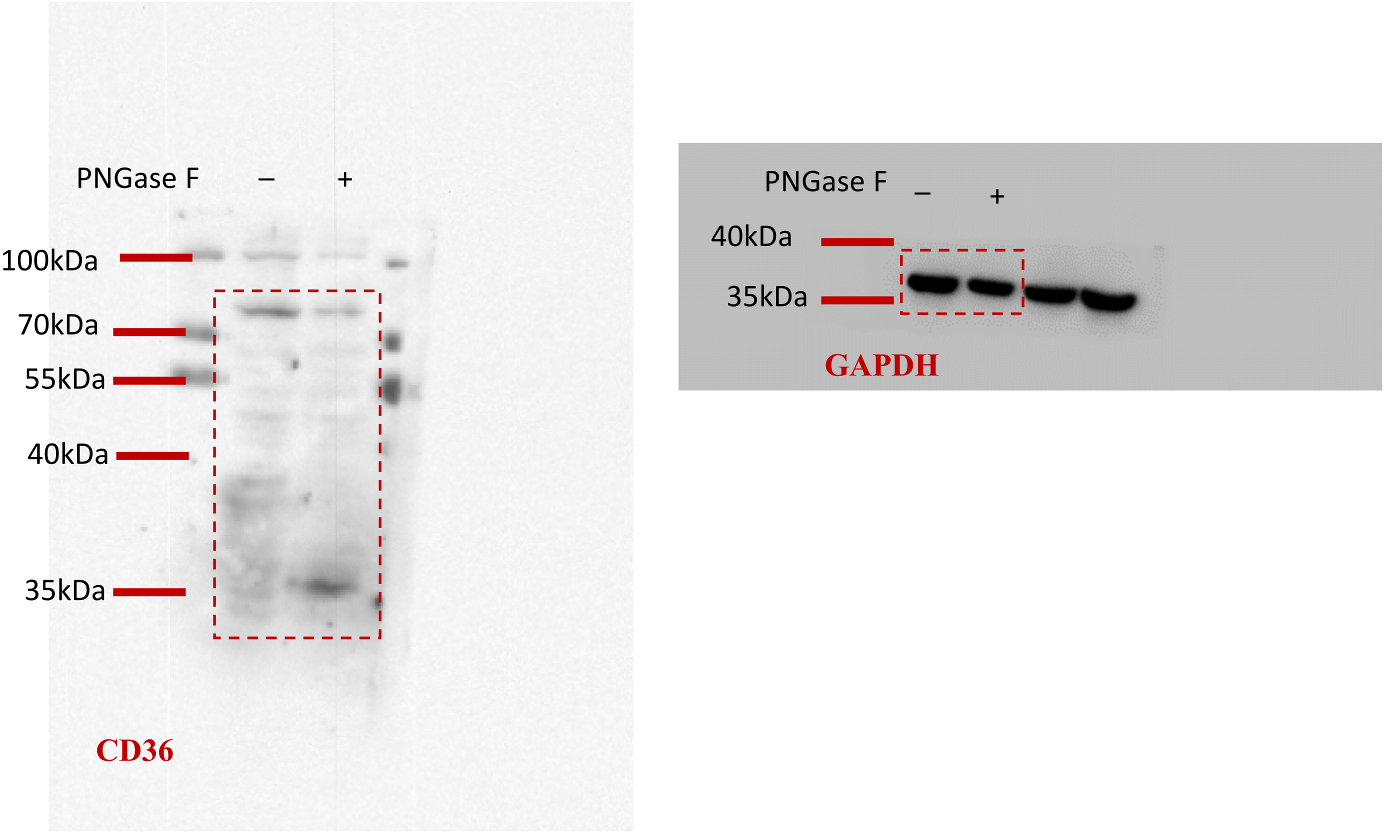

Figure 5 B

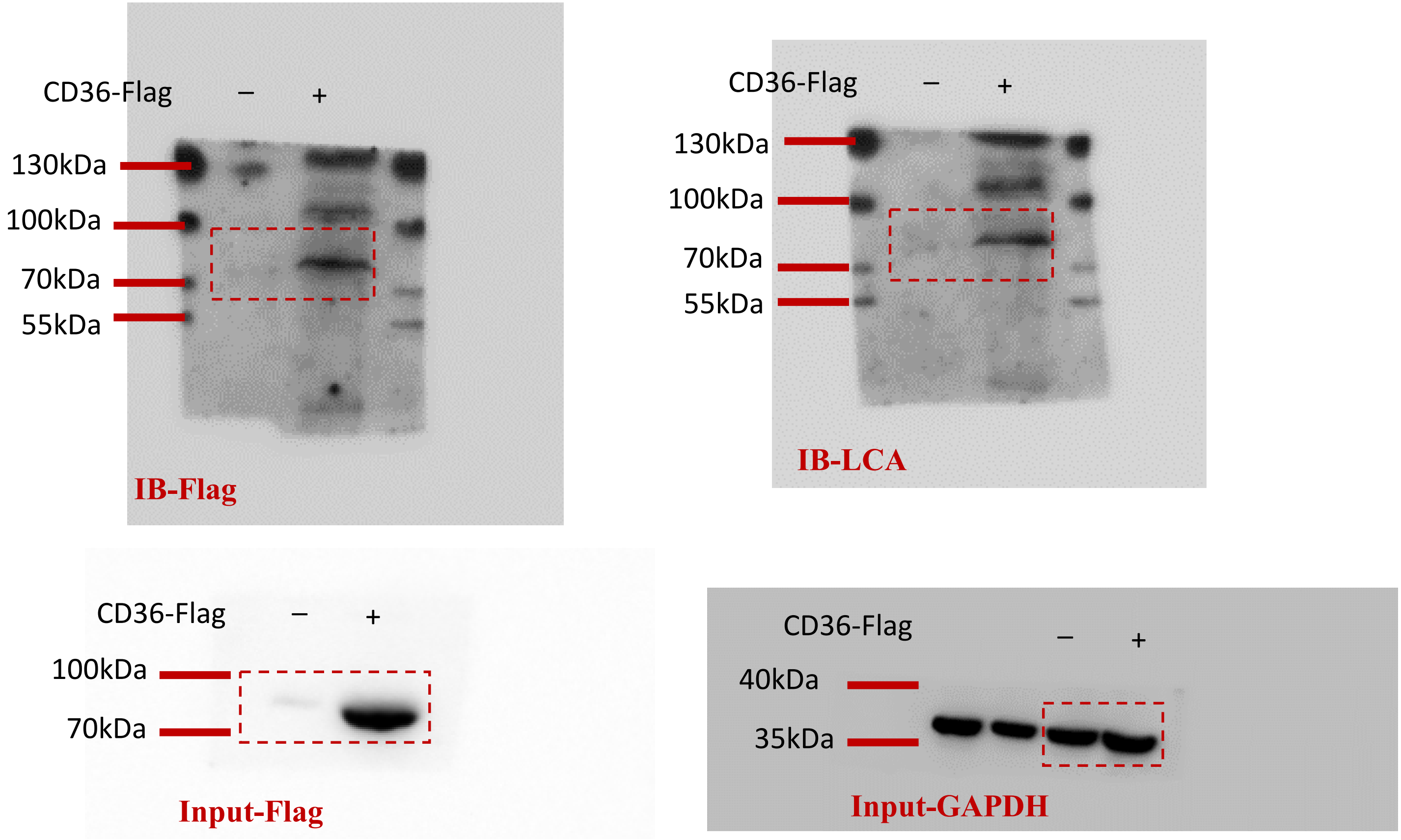

Figure 6 B

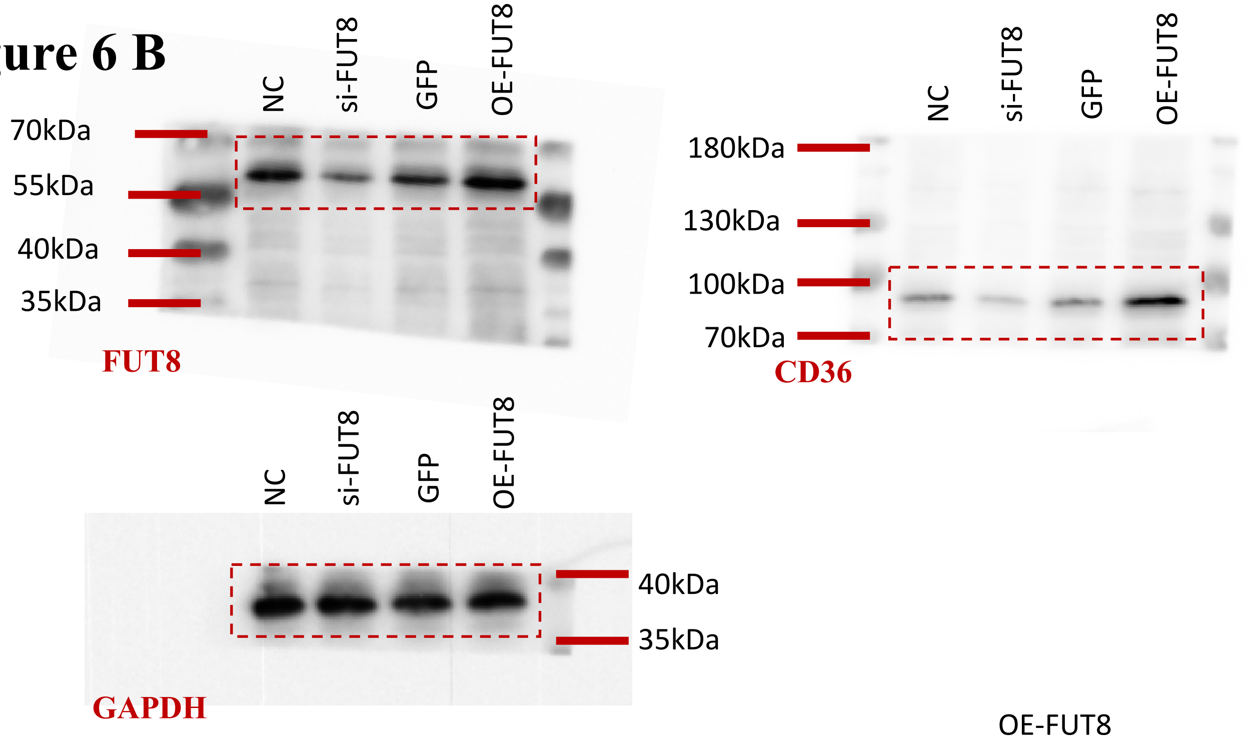

Figure 6 D

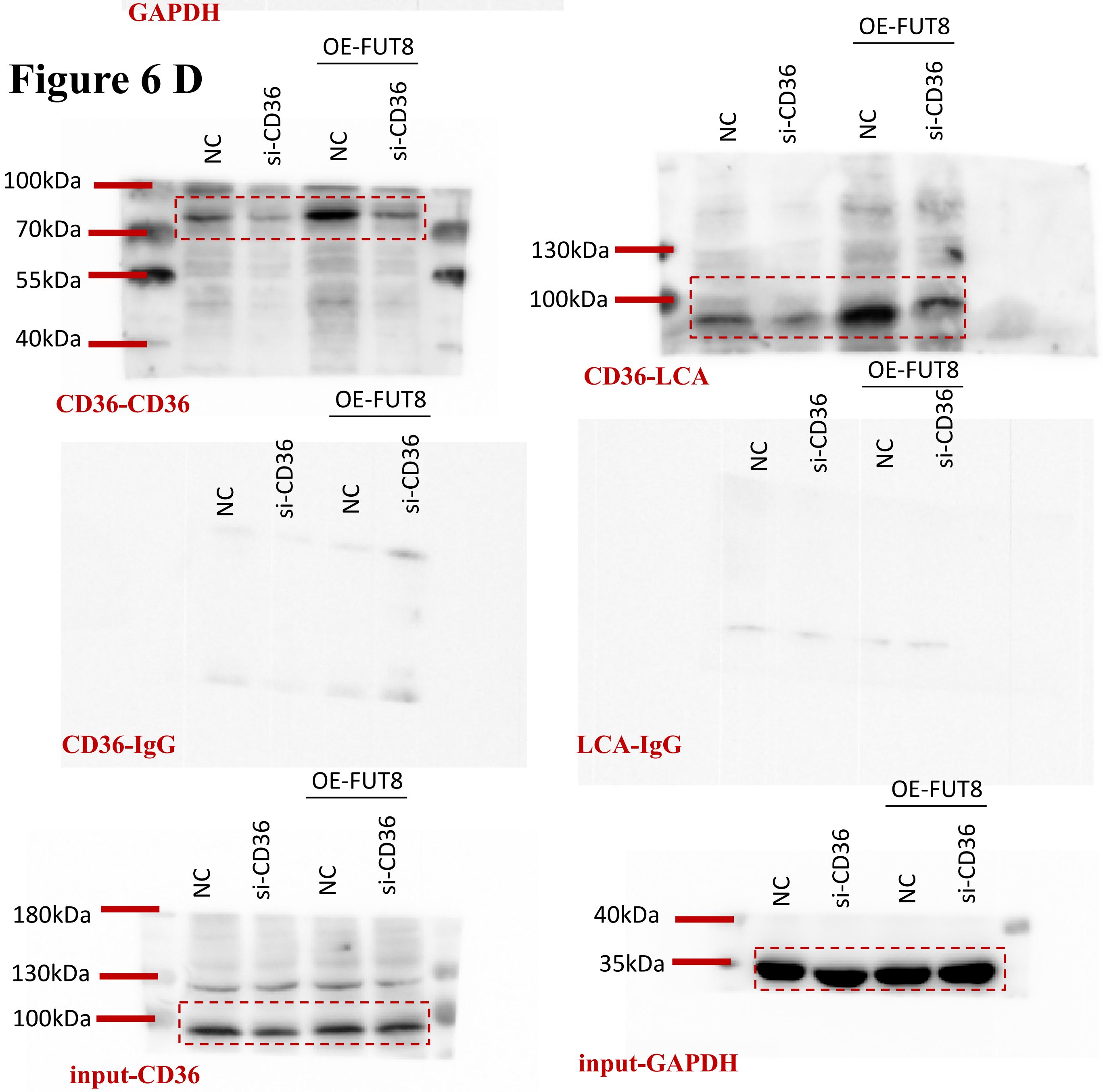

Figure 7 F

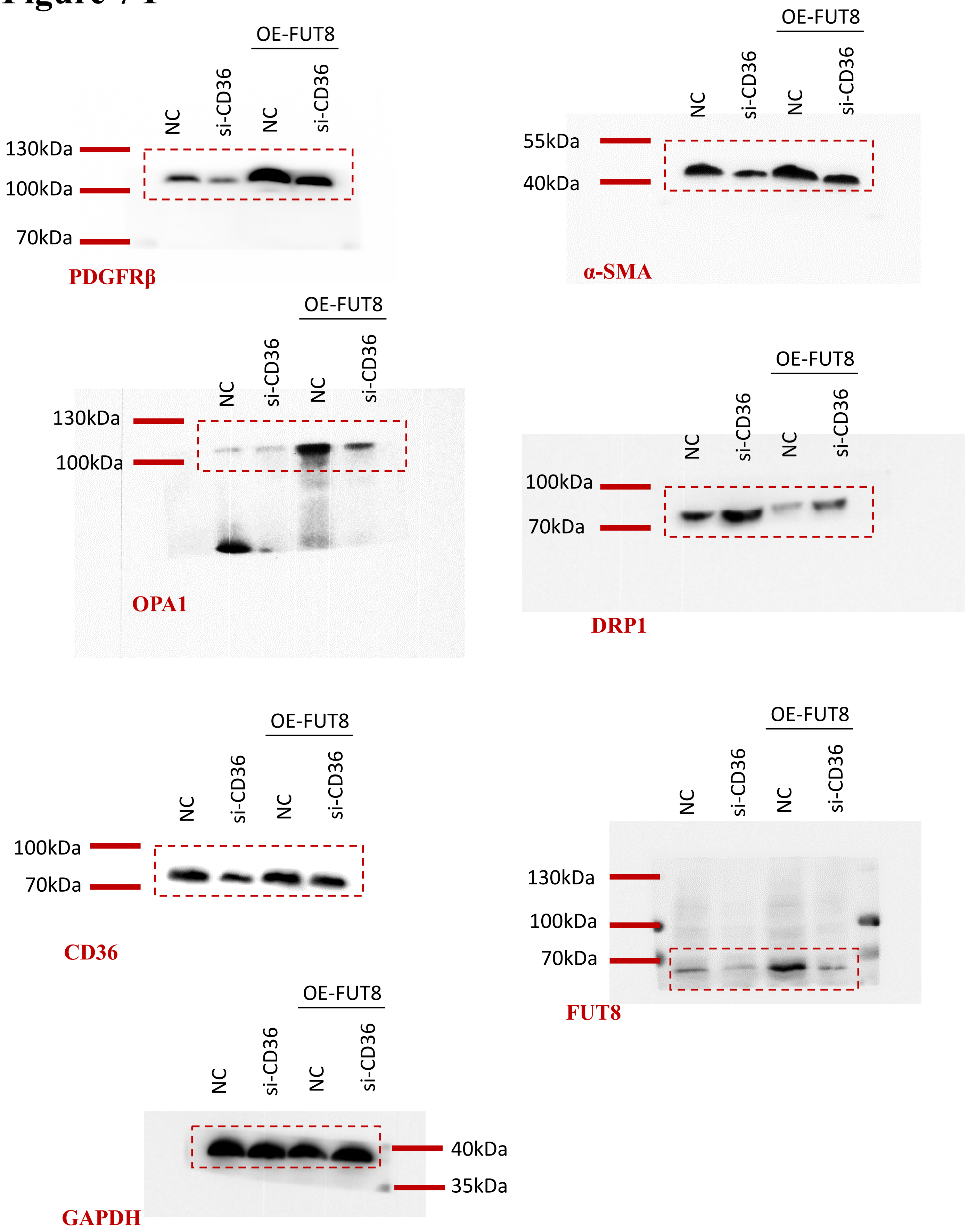

OE-FUT8
NC
si-CD36
NC
si-CD36
130kDa
100kDa
70kDa
PDGFRβ
OE-FUT8
NC
si-CD36
NC
si-CD36
55kDa
40kDa
α-SMA
OE-FUT8
NC
si-CD36
NC
si-CD36
130kDa
100kDa
OPA1
OE-FUT8
NC
si-CD36
NC
si-CD36
100kDa
70kDa
DRP1
OE-FUT8
NC
si-CD36
NC
si-CD36
100kDa
70kDa
CD36
OE-FUT8
NC
si-CD36
NC
si-CD36
130kDa
100kDa
70kDa
FUT8
OE-FUT8
NC
si-CD36
NC
si-CD36
40kDa
35kDa
GAPDH

Supplemental Figure E

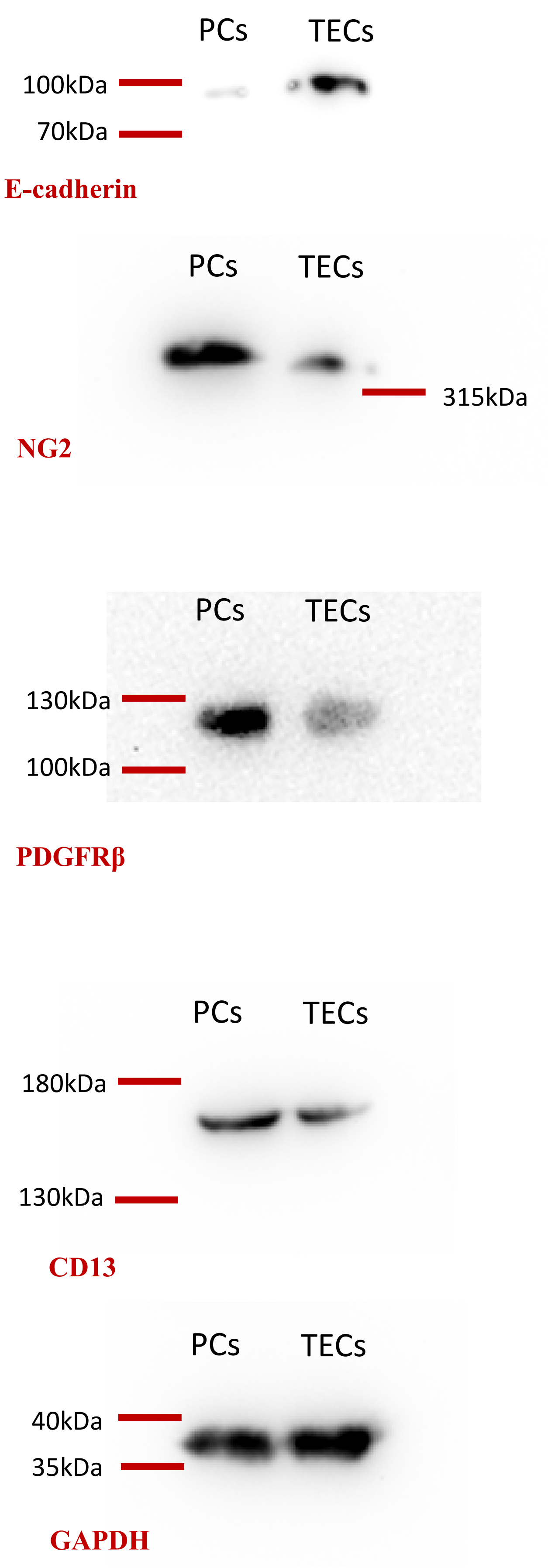

Supplement: Supplementary file 1 — Supplementary Material 1 [file 10020_2024_994_MOESM1_ESM.pdf]
