# Supplementary material for: FUT8 upregulates CD36 and its core fucosylation to accelerate pericyte-myofibroblast transition through the mitochondrial-dependent apoptosis pathway during AKI-CKD
Source: Mol Med. 2024 Nov 20;30:222. doi: 10.1186/s10020-024-00994-6 (PMC11577590; doi:10.1186/s10020-024-00994-6)
Supplement: Supplementary file 2 — Supplementary Material 2 [file 10020_2024_994_MOESM2_ESM.pdf]

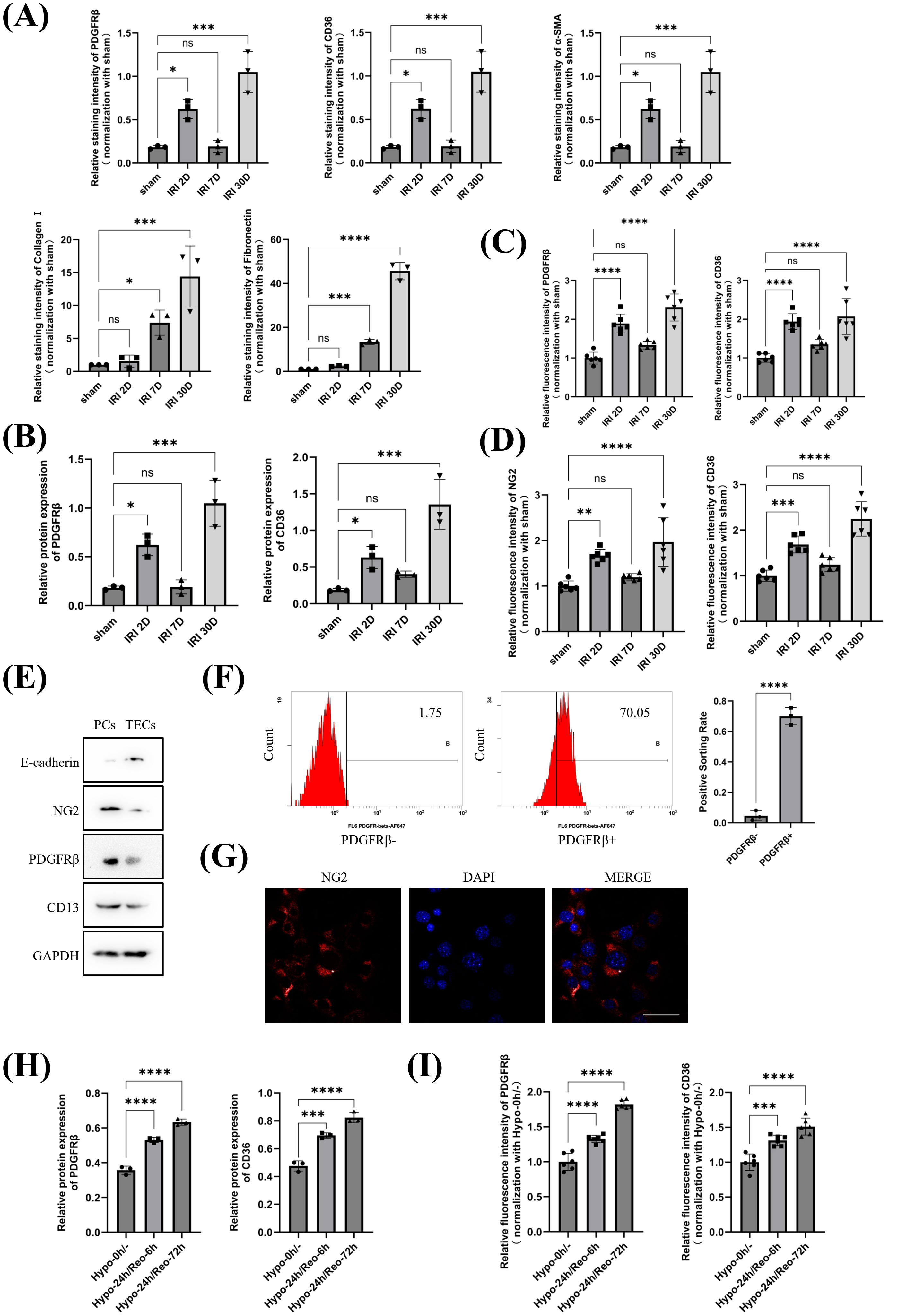

(A)
Relative staining intensity of PDGFRβ (normalization with sham)
Relative staining intensity of CD36 (normalization with sham)
Relative staining intensity of α-SMA (normalization with sham)
Relative staining intensity of Collagen Ⅰ (normalization with sham)
Relative staining intensity of Fibronectin (normalization with sham)
sham
IRI 2D
IRI 7D
IRI 30D
ns
(B)
Relative protein expression of PDGFRβ
Relative protein expression of CD36
(C)
Relative fluorescence intensity of PDGFRβ (normalization with sham)
Relative fluorescence intensity of CD36 (normalization with sham)
(D)
Relative fluorescence intensity of NG2 (normalization with sham)
Relative fluorescence intensity of CD36 (normalization with sham)
(E)
PCs TECs
E-cadherin
NG2
PDGFRβ
CD13
GAPDH
(F)
Count
1.75
70.05
FL6 PDGFR-beta-AF647
PDGFRβ-
PDGFRβ+
Positive Sorting Rate
(G)
NG2
DAPI
MERGE
(H)
Relative protein expression of PDGFRβ
Relative protein expression of CD36
Hypo-0h/-
Hypo-24h/Reo-6h
Hypo-24h/Reo-72h
(I)
Relative fluorescence intensity of PDGFRβ (normalization with Hypo-0h/-)
Relative fluorescence intensity of CD36 (normalization with Hypo-0h/-)

Supplemental Figure. (A) Quantification of PDGFRβ, CD36, α-SMA, Collagen I and Fibronectin levels in IHC ($n = 3$). (B)Quantification of CD36 and PDGFRβ levels in WB ($n = 3$). (C) and (D) Quantify the average fluorescence intensity of PDGFRβ, NG2 and CD36 ($n=3$). In each biological replicate, two fields were assessed as technical replicates. (E) Representative western blotting images of E-cadherin, NG2, PDGFRβ and CD13 in extracted mice primary pericytes (PCs) and tubule epithelial cells (TECs). (F) The application of a 42% Percoll solution resulted in a significant increase in the positive isolation rate. (G) Representative immunofluorescence staining images of NG2 (red) in mice primary pericytes (bar = 50 μm). (H) Quantification of CD36 and PDGFRβ levels ($n = 3$). (I) Quantify the average fluorescence intensity of CD36 and PDGFRβ ($n = 3$). In each biological replicate, two fields were assessed as technical replicates. ns, no difference; *$p < 0 .05$; **$p < 0.01$; ***$p < 0.001$; ****$p < 0.0001$, one-way ANOVA was used to determine statistical significance. Data were presented as mean ± SD.
